# ChIP-seq in steatohepatitis and normal liver tissue identifies candidate disease mechanisms related to progression to cancer

**DOI:** 10.1186/1755-8794-6-50

**Published:** 2013-11-08

**Authors:** Madhusudhan Bysani, Ola Wallerman, Susanne Bornelöv, Kurt Zatloukal, Jan Komorowski, Claes Wadelius

**Affiliations:** 1 Science for Life Laboratory, Department of Immunology, Genetics and Pathology, BMC, Uppsala University, PO BOX 815, Uppsala, SE 751 08, Sweden; 2 Science for Life Laboratory, Department of Cell and Molecular Biology, BMC, Uppsala University, Uppsala, Sweden; 3 Institute of Pathology, Medical University of Graz, Graz, Austria; 4 Interdisciplinary Centre for Mathematical and Computational Modelling, University of Warsaw, Warsaw, PL-02-106, Poland; 5 Science for Life Laboratory, Department of Medical Biochemistry and Microbiology, BMC, Uppsala, Sweden

**Keywords:** ChIP-seq, Tissue samples, Steatohepatitis, Cancer networks

## Abstract

**Background:**

Steatohepatitis occurs in alcoholic liver disease and may progress to liver cirrhosis and hepatocellular carcinoma. Its molecular pathogenesis is to a large degree unknown. Histone modifications play a key role in transcriptional regulations as marks for silencing and activation of gene expression and as marks for functional elements. Many transcription factors (TFs) are crucial for the control of the genes involved in metabolism, and abnormality in their function may lead to disease.

**Methods:**

We performed ChIP-seq of the histone modifications H3K4me1, H3K4me3 and H3K27ac and a candidate transcription factor (USF1) in liver tissue from patients with steatohepatitis and normal livers and correlated results to mRNA-expression and genotypes.

**Results:**

We found several regions that are differentially enriched for histone modifications between disease and normal tissue, and qRT-PCR results indicated that the expression of the tested genes strongly correlated with differential enrichment of histone modifications but is independent of USF1 enrichment. By gene ontology analysis of differentially modified genes we found many disease associated genes, some of which had previously been implicated in the etiology of steatohepatitis. Importantly, the genes associated to the strongest histone peaks in the patient were over-represented in cancer specific pathways suggesting that the tissue was on a path to develop to cancer, a common complication to the disease. We also found several novel SNPs and GWAS catalogue SNPs that are candidates to be functional and therefore needs further study.

**Conclusion:**

In summary we find that analysis of chromatin features in tissue samples provides insight into disease mechanisms.

## Background

Alcoholic steatohepatitsis (ASH) is a chronic liver disease that develops in approximately 20% of heavy drinkers. ASH leads to characteristic morphological alterations in the liver, such as accumulation of fat (steatosis), ballooning of hepatocytes, appearance of hepatocytic protein aggregates (Mallory Denk Bodies), necrosis and apoptosis, fibrosis, inflammation, and cholestasis. These alterations are similar to steatohepatitis that develops in patients without alcoholic liver diseases (non-alcoholic steatohepatitis; NASH) in the context of obesity, diabetes and the metabolic syndrome [1]. The similar morphological alterations in ASH and NASH suggest common pathophysiological mechanisms that still have to be characterized. Furthermore it is still unclear whether simple steatosis of the liver which is a relatively benign and in principle reversible disease progresses to steatohepatitis which further progresses to liver cirrhosis and eventually to cancer, or whether steatosis and steatohepatitis (ASH or NASH) are two different disorders [2]. A study on the role of alcohol metabolism in the pathogenesis of NASH found that the alcohol dehydrogenase genes, catalase genes, cytochrome P450 2E1 (*CYP2E1*) and aldehyde dehydrogenase have an increased level of transcription in NASH patients compared to controls [3]. Genome-wide association studies (GWAS) on African American and European American individuals showed that the ‘G’ allele of the rs738409 SNP in the *PNPLA3* gene was strongly associated with increased hepatic fat levels and with hepatic inflammation [4]. Another GWA study using variance components methods showed significant association with histological NAFLD and variants in or around the genes of *NCAN*, *GCKR* and *LYPLAL1*[5].

Chromatin Immunoprecipitation coupled with high throughput sequencing (ChIP-seq) allows the study of binding sites for TFs or sites of modified histones to be identified in a single experiment on a genome-wide scale. In this study, we used bio-bank samples from patients with steatohepatitis and compared it to samples from healthy controls. N-terminal modifications of histones may change the chromatin accessibility to transcription and they are associated with activation and silencing of the genes [6]. We compared the histone modification patterns in liver samples between patients with ASH and controls using ChIP-seq for three marks associated with active genes. Histone 3 lysine 4 tri-methylation (H3K4me3) is associated to promoters while Histone 3 lysine 4 mono-methylation (H3K4me1) is associated to enhancers and lysine 27 acetylation (H3K27ac) are found both at active enhancers and promoters. To our knowledge, this is the first epigenetic study on these three histone marks of steatohepatitis patients. USF1 is a transcription factor in the helix-loop-helix leucine zipper family that binds the E-box sequence CA[C/T]GTG at many genes important for lipid and cholesterol metabolism [7]. Two SNPs (rs2073658 and rs3737787) in *USF1* have been associated to the disease familial combined hyperlipidemia [8] and furthermore, these SNPs have been have been associated to an increased risk of type 2 diabetes in a case control association study on Dutch Caucasians [9]. We therefore hypothesized that USF1 may play a role in steatohepatitis and studied its binding sites using ChIP-seq on biobank samples.

We find that the pattern of histone modifications was similar when comparing normal liver tissue and alcoholic steatohepatitis. However, in differentially enriched regions we find genes known to be involved in the disease as well as new genes that are candidates to contribute to the pathology. In particular, in the patient we find genes in cancer pathways suggesting that the tissue is en route to a malignancy, a known complication to the disease.

## Methods

### Tissue samples

Frozen liver tissue samples from three patients with ASH and three controls without chronic liver diseases (patients who underwent liver surgery because of liver metastasis from colon cancer) were obtained from Graz bio-bank, Medical university of Graz, Austria. The first control and patient set was used to map USF1, the second set was used to study the histone modifications and a third set along with the above two sets was used for RNA expression analysis by qRT-PCR.

### Chromatin immunoprecipitation

Frozen liver tissues were chopped into small pieces and crosslinked with a final concentration of 0.37% formaldehyde for 10 minutes and the crosslinking was stopped by adding glycine to a final concentration of 0.125 M. Crosslinked tissue was washed by 1X PBS and cell lysis buffer with protease inhibitors (PIs) was added and nuclei were prepared by using a dounce homogenizer. RIPA buffer (1X PBS, 1% NP-40, 0.1% SDS, 0.5% Sodium deoxycholate, 0.004% sodium azide) with PIs was added to the nuclei and chromatin was sonicated to 100-300 bp fragments using a BioRuptor (Diagenode). After preclearing with Protein-G-Agarose beads (Roche), chromatin was incubated with antibody overnight. Antibodies were from Santa Cruz Biotechnology for USF1 (sc-229) and from Abcam for histone modifications (ab8895, ab8580 and ab4729). Protein-G agarose beads were added and incubated for two hours and then the chromatin-antibody-bead complex was washed four times with RIPA buffer, once with ChIP wash buffer 2 (0.01 M Tris–HCl (pH 8), 0.25 M LiCl, 0.001 M EDTA, 1% NP-40, 1% Sodium deoxycholate) and once with TE buffer. The protein-DNA complex was eluted in IP elution buffer (0.1 M NaHCO_3_ and 1% SDS) with vigorous shaking of beads at room temperature and crosslinks were reversed at 65°C with 0.3 M Sodium chloride and RNase A for 6 hours followed by 45°C overnight incubation with Proteinase K. The DNA was extracted by phenol/chloroform and ethanol precipitation and the pellet was dissolved in water. The ChIP DNA enrichment was verified by semi-quantitative PCR using two known positive and two negative targets.

### SOLiD fragment library preparation

SOLiD fragment libraries were prepared according to the manufacturer’s protocol. ChIP DNA fragments were end repaired and ligated with adapters. Ligated fragments were amplified using adapter specific primers and size selected on Invitrogen’s flash gel. Approximately 200-300 bp fragments (including adapters) were collected. These libraries were sequenced on the ABI SOLiDV3 platform.

### Illumina library preparation

ChIP enriched DNA was end repaired using end repair kit (Epicentre) at room temperature for 45 minutes, ‘A’ base was added to the 3′ ends of the fragments using Klenow (3′-5′ exo-) (NEB) by incubating at 37°C for 30 minutes. These fragments were ligated with Illumina adapters using quick ligase kit (NEB). Adapter ligated fragments were enriched by PCR with *Pfu* ultra high sensitivity master mix (Agilent) and adapter specific primers. The PCR product was size-selected on 2% TAE agarose gel and purified with Qiagen gel extraction columns. Qiagen MinElute columns were used for purification after end repair and A-tailing and AMPure XP beads were used to purify and size-select the ligation mix before amplification. Sequencing was done on Illumina Hiseq platform using paired-end technology. Quality and size range of libraries for both the platforms were checked on Bioanalyzer (Agilent technologies) before sequencing.

### Sanger sequencing

Primers were designed for the SNPs located near a motif for USF1 in peaks with potential differential enrichment. USF1 enriched ChIP DNA and genomic DNA of patient and control was amplified and purified and PCR products were sequenced using BigDye terminator v3.1 (Applied Bio system) and capillary electrophoresis on a ABI 3730XL DNA analyzer. Obtained sequences were analyzed by using Sequencher software.

### Quantitative PCR

New replicate of ChIP was performed with the tissues from the same individuals using anti-USF1 antibody and primers for qPCR were designed around the USF1 enriched regions and the regions without enrichment. qPCR in triplicates was used to measure enrichment by comparing to a standard curve obtained for each primer by serial dilution of input DNA and to a background level calculated as the average + 2*standard deviation of the values for negative regions. Enrichment of more than two-fold over this background level was considered as positive.

### RNA Preparation, Synthesis of cDNA and qRT-PCR

Total RNA was extracted from tissue samples using mammalian total RNA extraction kit (Sigma Aldrich). First Strand cDNA was synthesized from 5 μg of total RNA using the Maxima First Strand cDNA synthesis kit (Thermo Scientific). The reaction was performed at 50°C for 30 minutes and terminated by incubating at 85°C for 5 minutes. qPCR was performed with cDNA in triplicates and normalized to the house keeping genes GAPDH, β-Actin and RSP18.

### ChIP-seq data analysis

The SOLiD 50 bp reads for USF1 were aligned to the hg18 reference genome using BFAST (v. 6.4) with the -A 3 setting which excludes reads with more than one best scoring alignment. We further removed all alignments with more than five mismatches in the first 40 colors and a mapping quality below 20. Only one read per start position was retained. Reads were extended *in silico* to the average fragment length (170 bp) and the overlaps of the extended fragments were calculated throughout the genome. Thresholds for significance (more than 5 and 10 reads for ASH and control respectively) were set based on the number of reads used for peak calling and the enrichment compared to background to get comparable datasets for patient and control, and peaks of enrichment higher than the thresholds were identified. The center position with highest overlap count was identified in each peak and used as basis for motif discovery and location analysis. *De novo* motif analysis was done using the online version of MEME-ChIP (http://meme.nbcr.net/meme/cgi-bin/meme-chip.cgi) based on the 500 highest and lowest peaks for each dataset.

The Illumina reads for histone modifications were aligned to hg18 using bwa [10]. Duplicates were removed using Picard tools (http://picard.sourceforge.net). MACS was used to call peaks, and for differential enrichment analysis SICTIN [11] was used to count reads in the peak regions and the counts were normalized by the total number of mapped read. The difference in counts between ASH and control was used to rank genes as higher in patient or higher in control. For genomic distributions, all histone modification peaks with an overlap towards the TSS or the 3′ end of the genes were identified. All 37 175 genes annotated in the Ensembl were considered. Intragenic peaks were defined as a peak with any overlap to a gene, and intergenic peaks as those without any overlap. The statistics was calculated for the three histone modifications and two states (higher in patient than in control, or higher in control than in patient), which gave six categories in total. Regions with more than 80% overlap to the simpleRepeat track downloaded from the UCSC Genome Browser were removed.

### Footprints over TSS

SICTIN *build binary* was used to transform the bed files into a binary format and *make footprint* to count the average number of reads for a region of +/− 1000 bp from the TSS. The gene annotations were collected from the Ensembl [12] system (*H.sapiens* 54_36p). Only the 19 950 genes annotated as protein coding were used. Each histone modification was normalized to have the same total sum over all 2001 positions, and then scaled so that the maximal observed enrichment had a value of 1.

### Gene ontology

For gene ontology enrichment analysis we identified the 1000 peaks with highest differential enrichment in patient and in control for each histone modification and for each peak selected the gene with the closest TSS. For USF1 we used the nearest gene for all peaks.

### SNP calling

Aligned reads from all three histone modifications from the same individual were merged to a single file and SNP calling was done using samtools and bcftools. SNPs in regions with sequencing depth > 100 were excluded to avoid repetitive sequences. SnpSift [13] was used to remove SNPs with quality < 50. Annotated SNPs were separated from possibly novel SNPs with annovar [14] and dbSNP version 129. Possibly novel SNPs with at least ten reads in the peak regions were identified in the patient by additionally filter using dnpSNP version 132. Those were annotated using SnpEff [13].

The annotated SNPs in patient and control were compared to “A Catalog of published Genome-Wide Association Studies” (http://www.genome.gov/26525384) and SNPs in either sample that had been reported in GWASs related to certain keywords (diabetes, fasting plasma glucose, hepatitis, hepatocellular carcinoma, insulin, LDL cholesterol, lipid metabolism, liver, obesity, triglycerides) were identified. In the GWAS database, only the strongest associations are reported when there are multiple hits in the same region. In order to also identify the weaker hits in the samples, we searched for SNPs in high LD with GWAS SNPs using the SNAP proxy search (http://www.broadinstitute.org/mpg/snap/ldsearch.php) with data from the CEU panel in the 1000 Genomes Pilot project, using an r^2^ threshold of 0.8 and a distance limit of 500 kb. The SNPs were annotated with whether they had overlap to a HM peak only in control, only in patient, in both, or in none.

## Results

### ChIP-sequencing and USF1 peaks

In the ChIP-seq experiments we used Illumina HiSeq for analysis of H3K4me1, H3K4me3 and H3K27ac and Life Technologies SOLiD platform for USF1. After filtering of duplicate and ambiguously mapped reads 5 – 24.7 M reads remained for peak calling (Tables 1 and 2).

**Table 1:** USF1 ChIP-seq reads and peak calls

	**Unique aligned reads**	**Cut-off (peak height)**	**Peaks**	**Peaks with motif**
**Control**	17 M	10	2054	895
**ASH**	5 M	5	1766	948

**Table 2:** Histone modification ChIP-seq reads and peak calls

	**Control**				**ASH**			
	**Total reads**	**Aligned reads**	**Dup. rate**	**Peaks**	**Total reads**	**Aligned reads**	**Dup. rate**	**Peaks**
**H3K4me1**	17.8 M	11.4 M	0.5%	10255	27.6 M	19.6 M	4.8%	10673
**H3K4me3**	14.5 M	8.7 M	0.8%	14667	28.7 M	24.7 M	2.5%	18357
**H3K27ac**	12.7 M	9.4 M	0.5%	11655	27.2 M	21 M	2.6%	12384

**Figure 1:**
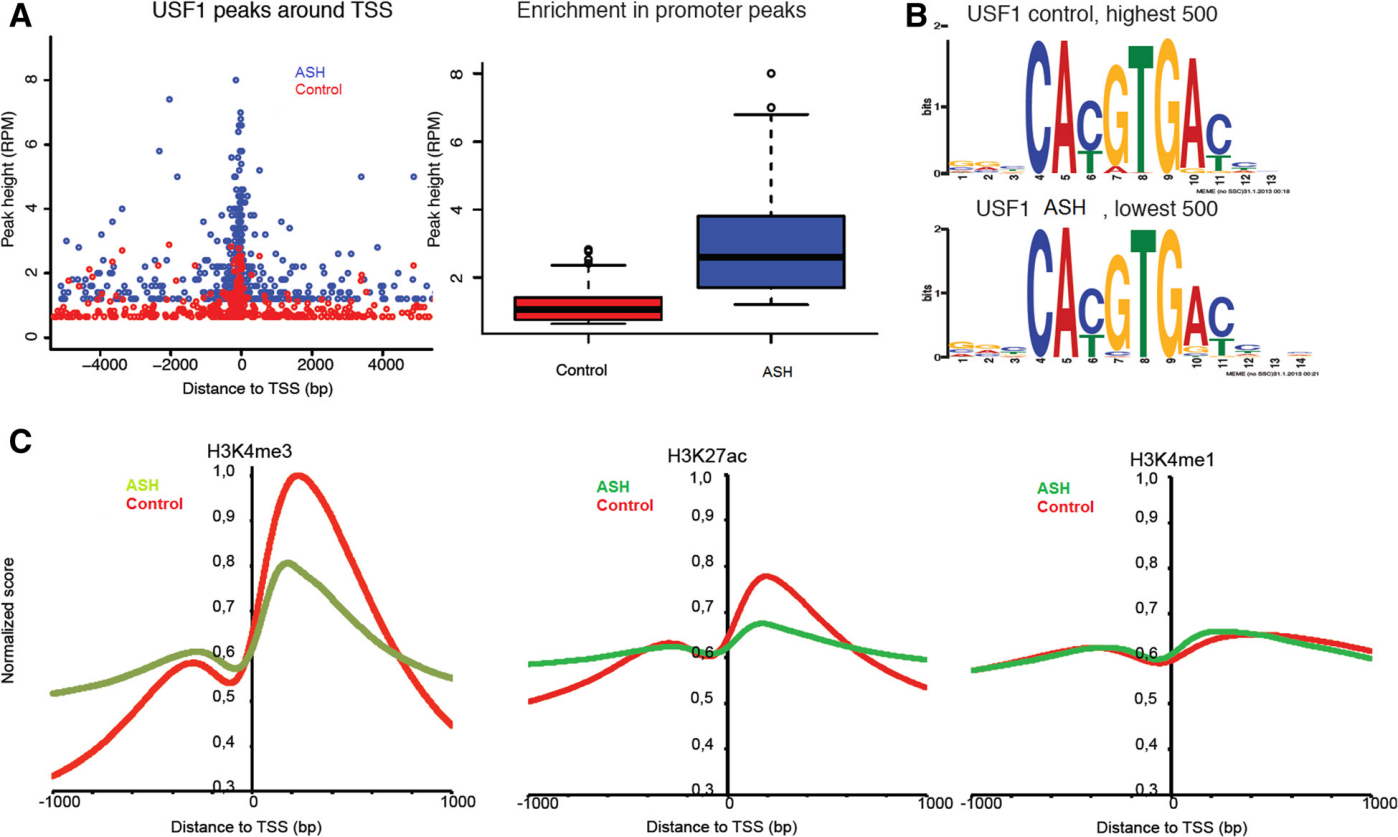
**ChIP-seq peak distributions. A)** Distribution and peak heights (left) of USF1 peaks around the TSS and the enrichment at promoters (right). Peak heights are reported in reads per million (RPM). **B)** The USF1 motif was identified in both high and low USF1 peaks. **C)** Foot prints in 2 kb windows of TSS for the Histone marks H3K4me1, H3K4me3, H3K27ac.

The USF1 peak lists were filtered for regions enriched also in input chromatin and the enrichment threshold was set to account for the different number of reads in the samples, which gave a set of 2054 enriched peaks for control and 1766 peaks for ASH (Table 1 and Additional file 1). The genome-wide distribution of peaks was similar to what have been seen in the HepG2 cell line [7] with a marked enrichment for peaks immediately upstream of the TSS for both samples but with a better enrichment obtained for the patient sample (Figure 1A). The USF1 motif with the E-box recognition sequence CA[C/T]GTG could be identified *de novo* from both datasets and was enriched in both the highest and lowest peaks (Figure 1B and Additional file 2: Figure S7), which indicates that the peak lists to a large extent represents direct USF1 bindings. In all, we found 895 (44%) peaks for the control and 948 (54%) peaks for the ASH sample with at least one match to the CAC[G/A]TG sequence within 50 bps of the peak center (Table 1).

To further validate the ChIP-seq results we tested thirteen positive and four negative sites with qPCR on a replicate ChIP sample and found a good correlation (R^2^ = 0.41 for the control and 0.62 for the patient sample) between the ChIP-seq and qPCR enrichment indicating good quality in the data (Additional file 2: Figure S1).

### Detection and analysis of differentially enriched regions for histone modifications

To identify differentially modified regions, we first identified enriched regions using the MACS peak finder, which gave 10 317–18 358 peaks for the different samples (Table 2). The overall patterns of histone modifications were strongly correlated between patients and controls (Additional file 2: Figure S2), as can be expected for samples from the same tissue type. Considering that the number of peaks may depend on the number of reads, we used a fixed number of peaks from each sample for further analysis. Using the fewest number of peaks found in a sample, rounded by thousands, the 10 000 highest ranked (P-value) peaks were kept for each sample. The normalized read counts in the peak regions were calculated for both samples for each peak region identified in either ASH patient or control, and peaks were ranked according to the difference in enrichment. The numerical distributions of differences were quite skewed and different for the different modifications, i.e. H3K4me3 and H3K27ac had higher signals in patient than in control, whereas H3K4me1 had higher signal in control than in patient (Additional file 2). To avoid introducing any arbitrary assumptions about the data, for each histone modification we selected the 1000 regions with the highest difference in each direction as differentially enriched regions. For each differential region the Ensembl database was used to identify the closest gene (Additional file 3).

**Figure 2:**
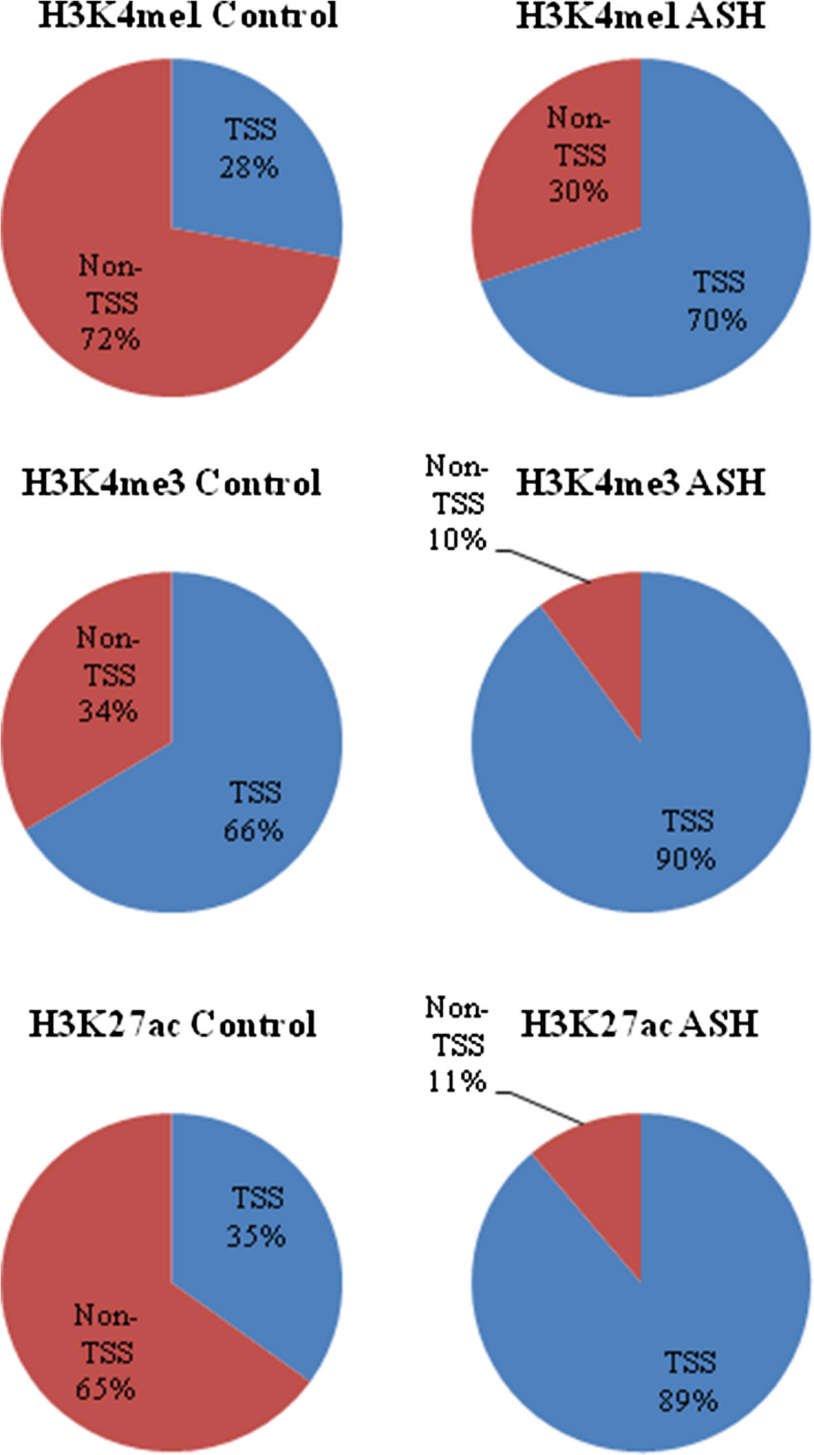
**Histone modification peaks at TSS.** Percentage of differentially enriched peaks identified at 2 kb of TSS and non TSS sites for H3K4me1, H3K4me3 and H3K27ac in control and ASH samples.

From the ChIP-seq enrichment profiles we generated footprints within 2 kb windows of transcription start sites (TSS) for the three histone modifications (Figure 1C). As expected from previous studies, we have observed double peaks centered on the TSS for all three modifications with the strongest enrichment for H3K4me3 and H3K27ac as these marks are mainly promoter-associated histone marks of active genes [15]. The double peaks could be an indication of bidirectional promoters [7]. We have previously observed this type of bidirectional pattern in HepG2 cells for H3K4me3 mark [16]. We also looked into genomic localization of differentially enriched histone modifications across the genome, i.e. at +/− 2 kb of TSS and non-TSS regions (Figure 2). As expected, the majority of the H3K4me3 peaks are located within 2 kb of TSS, whereas the peaks for H3K4me1 and H3K27ac are distributed both at TSS and non-TSS regions (Figure 2). The majority of identified genes had peaks for both control and ASH, with around 1600–3000 genes containing a significant peak only in patient or in control (Additional file 2: Figure S3). We further compared the lists with differentially enriched histone modification peaks to the USF1 binding sites but did not observe any significant difference in USF1 signal for these genes. This indicates that USF1 is not a major contributor to differential gene regulation between the steatohepatitis patients and controls.

### Gene ontology prediction of genes involved in liver metabolism and cancer

We used the web based functional annotation tool (DAVID) (http://david.abcc.ncifcrf.gov/summary.jsp) [17] for gene ontology (GO) for genes with USF1 binding sites and for genes with differential histone modifications and identified many groups of genes that are involved in different liver specific mechanisms and pathways. GO analysis of the highest USF1 peaks did not show any major difference between the individuals. We combined the gene lists with differential histone modifications for each sample (Additional file 4) and found some of the most significantly enriched pathways that were detected in the control but not in the patient to be fatty acid metabolism and pyruvate metabolism (Table 3). Importantly, in the patient data set we identified cancer specific pathways and pathways for basal cell carcinoma. We further looked at the role of these genes in hepatocellular (http://liverome.kobic.re.kr/index.php) [18] for liver-cancer related gene lists and found that some of the genes present in these pathways were known to have changed their expression in liver cancer (Table 4). This indicates that the gene expression pattern has changed from a normal liver metabolism to a state that is more cancer-like in the patient, and that the ASH in this patient may be on the way to progress into HCC.

**Table 3:** Pathways identified using the genes associated with ChIP-seq histone modification peaks for ASH and control

**KEGG pathway**	**ENSEMBL_GENE_ID**	**Gene name**
***Control***		
Glycine, serine and threonine metabolism	ENSG00000092621	Phosphoglycerate dehydrogenase
	ENSG00000131471	Amine oxidase, copper containing 3 (vascular adhesion protein 1)
	ENSG00000168237	Glycerate kinase
	ENSG00000182199	Serine hydroxymethyltransferase 2 (mitochondrial)
	ENSG00000069535	Monoamine oxidase B
	ENSG00000145692	Betaine-homocysteine methyltransferase
	ENSG00000160200	Cystathionine-beta-synthase
	ENSG00000145020	Aminomethyltransferase
	ENSG00000023330	Aminolevulinate, delta-, synthase 1
	ENSG00000172482	Alanine-glyoxylate aminotransferase
Pyruvate metabolism	ENSG00000076555	Acetyl-Coenzyme A carboxylase beta
	ENSG00000168291	Pyruvate dehydrogenase (lipoamide) beta
	ENSG00000154930	Acyl-CoA synthetase short-chain family member 1
	ENSG00000166816	Lactate dehydrogenase D
	ENSG00000173599	Pyruvate carboxylase
	ENSG00000063854	Hydroxyacylglutathione hydrolase
Fatty acid metabolism	ENSG00000127884	Enoyl Coenzyme A hydratase, short chain, 1, mitochondrial
	ENSG00000162365	Cytochrome P450, family 4, subfamily A, polypeptide 22
	ENSG00000105607	Glutaryl-Coenzyme A dehydrogenase
	ENSG00000151726	Acyl-CoA synthetase long-chain family member 1
	ENSG00000187048	Cytochrome P450, family 4, subfamily A, polypeptide 11
	ENSG00000196344	Alcohol dehydrogenase 7 (class IV), mu or sigma polypeptide
PPAR signaling pathway	ENSG00000083807	Solute carrier family 27 (fatty acid transporter), member 5
	ENSG00000186350	Retinoid X receptor, alpha
	ENSG00000165269	Aquaporin 7
	ENSG00000162365	Cytochrome P450, family 4, subfamily A, polypeptide 22
	ENSG00000151726	Acyl-CoA synthetase long-chain family member 1
	ENSG00000118137	Apolipoprotein A-I
	ENSG00000187048	Cytochrome P450, family 4, subfamily A, polypeptide 11
	ENSG00000140284	Solute carrier family 27 (fatty acid transporter), member 2
Steroid biosynthesis	ENSG00000052802	Sterol-C4-methyl oxidase-like
	ENSG00000109929	Sterol-C5-desaturase (ERG3 delta-5-desaturase homolog, S. cerevisiae)-like
	ENSG00000116133	24-dehydrocholesterol reductase
	ENSG00000001630	Cytochrome P450, family 51, subfamily A, polypeptide 1
***ASH***		
Pathways in cancer	ENSG00000108091	Coiled-coil domain containing 6
	ENSG00000168040	Fas (TNFRSF6)-associated via death domain
	ENSG00000197461	Platelet-derived growth factor alpha polypeptide
	ENSG00000143816	Wingless-type MMTV integration site family, member 9A
	ENSG00000100644	Hypoxia inducible factor 1, alpha subunit (basic helix-loop-helix transcription factor)
	ENSG00000177885	Growth factor receptor-bound protein 2
	ENSG00000006451	v-ral simian leukemia viral oncogene homolog A (ras related)
	ENSG00000133101	Cyclin A1
	ENSG00000157404	Similar to Mast/stem cell growth factor receptor precursor (SCFR) (Proto-oncogene tyrosine-protein kinase Kit)Ta
	ENSG00000175305	Cyclin E2
	ENSG00000168036	Catenin (cadherin-associated protein), beta 1, 88 kDa
	ENSG00000147889	Cyclin-dependent kinase inhibitor 2A (melanoma, p16, inhibits CDK4)
	ENSG00000033800	Protein inhibitor of activated STAT, 1
	ENSG00000145675	Phosphoinositide-3-kinase, regulatory subunit 1 (alpha)
	ENSG00000044115	Catenin (cadherin-associated protein), alpha 1, 102 kDa
	ENSG00000156427	Fibroblast growth factor 18
	ENSG00000135766	Egl nine homolog 1 (C. elegans)
	ENSG00000196591	Histone deacetylase 2
	ENSG00000138448	Integrin, alpha V (vitronectin receptor, alpha polypeptide, antigen CD51)
	ENSG00000108379	Wingless-type MMTV integration site family, member 3
	ENSG00000104290	Frizzled homolog 3 (Drosophila)
	ENSG00000125084	Wingless-type MMTV integration site family, member 1
	ENSG00000102678	Fibroblast growth factor 9 (glia-activating factor)
	ENSG00000185920	Patched homolog 1 (Drosophila)
	ENSG00000111186	Wingless-type MMTV integration site family, member 5B
	ENSG00000161958	Fibroblast growth factor 11
	ENSG00000128602	Smoothened homolog (Drosophila)
	ENSG00000138795	Lymphoid enhancer-binding factor 1
	ENSG00000053747	Laminin, alpha 3
	ENSG00000056558	TNF receptor-associated factor 1
	ENSG00000131759	Retinoic acid receptor, alpha
	ENSG00000099942	v-crk sarcoma virus CT10 oncogene homolog (avian)-like
	ENSG00000139687	Retinoblastoma 1
	ENSG00000007968	E2F transcription factor 2
Basal cell carcinoma	ENSG00000111186	Wingless-type MMTV integration site family, member 5B
	ENSG00000128602	Smoothened homolog (Drosophila)
	ENSG00000108379	Wingless-type MMTV integration site family, member 3
	ENSG00000138795	Lymphoid enhancer-binding factor 1
	ENSG00000168036	Catenin (cadherin-associated protein), beta 1, 88 kDa
	ENSG00000104290	Frizzled homolog 3 (Drosophila)
	ENSG00000125084	Wingless-type MMTV integration site family, member 1
	ENSG00000143816	Wingless-type MMTV integration site family, member 9A

**Table 4:** Expression profiles of liver-cancer associated genes

**ENSEMBL Gene ID**	**Gene name**	**Gene symbol**	**Regulation**	**Study references**
ENSG00000197461	Platelet-derived growth factor alpha polypeptide	PDGFA	Up	[19]
ENSG00000177885	Growth factor receptor-bound protein 2	GRB2	Up/Down	[19]
ENSG00000168036	Catenin (cadherin-associated protein), beta 1, 88 kDa	CTNNB1	Up/Down	[19]
ENSG00000147889	Cyclin-dependent kinase inhibitor 2A	CDKN2A	Up	[20]
	(melanoma, p16, inhibits CDK4)			
ENSG00000102678	Fibroblast growth factor 9 (glia-activating factor)	FGF9	Up	[21]
ENSG00000053747	Laminin, alpha 3	LAMA3	Up	[22-24]
ENSG00000139687	Retinoblastoma 1	RB1	Down	[25]

We have used another web based tool GORilla (http://cbl-gorilla.cs.technion.ac.il) [26] to further support our GO findings in DAVID. From the GORilla database, we found that the genes related to metabolism were enriched in both the patient and control. Surprisingly, the metabolism related genes had lower ChIP-seq peaks indicating lower gene expression in the patient (based on p-value) (Additional file 2: Figure S4) compared to control (Additional file 2: Figure S5). These findings further support our hypothesis that as the disease progresses, metabolism-related genes were down-regulated and cancer-related genes became activated and up-regulated.

**Figure 3:**
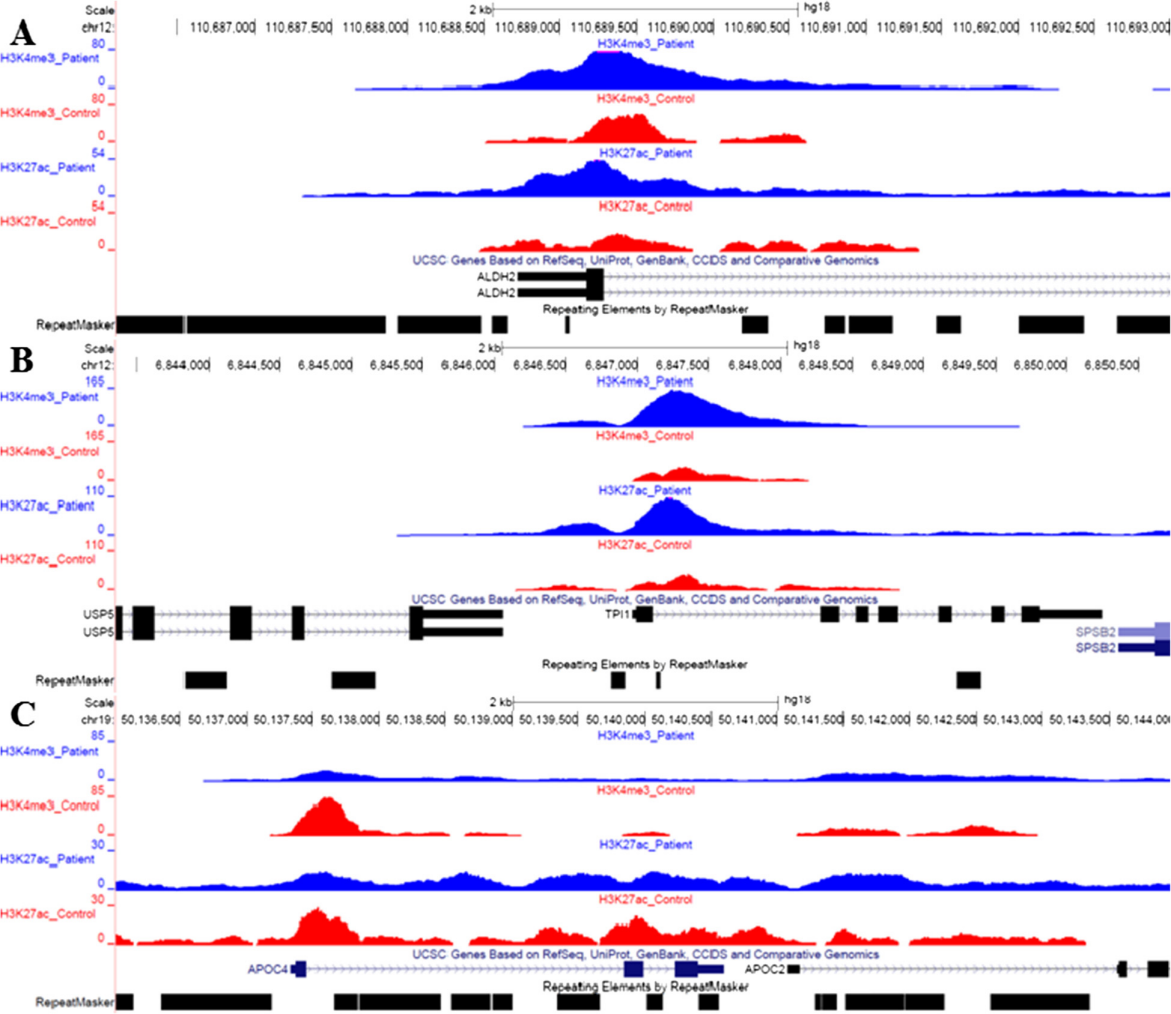
**ChIP-seq enrichment at selected sites.** Normalized ChIP-seq enrichment profiles (RPM) for the histone modifications H3K4me1, H3K4me3 and H3K27ac at the promoters of **A) ***ALDH2, ***B) ***TPI1* and **C) ***APOC4* genes.

Figure 3 shows the signal for three metabolism related genes with different histone modification patterns. Triosephosphate isomerase 1 (*TPI1)* catalyses the isomerisation of Glyceraldehyde 3 phosphate (*G3P)* and Dihydroxy acetone phosphate (*DHAP)* which is involved in glycolysis and in gluconeogenesis (Figure 3B). ALDH2 is an enzyme involved in alcohol metabolism. Higher expression of *ALDH2* has been observed in NASH patients than in normal liver [3] and we found stronger ChIP-seq peaks at the promoter of *ALDH2* in ASH patient than in control compatible with a common pathophysiological mechanisms in ASH and NASH (Figure 3A). The promoter of the Apolipoprotein C4 gene (*APOC4*) had lower signal for activating histone modifications in the patient than in the control suggesting that the gene is inactivated in the patient (Figure 3C). APOC4 *is* a key regulator in lipid transport [27] and a further support for the finding that genes in metabolism may be down-regulated as the disease progress.

We have investigated the histone modification pattern of the genes involved in alcohol metabolism and alcohol-related liver disease in the patient and control. There were stronger peaks in the patient than the control for the histone modifications at the promoters for Monocyte differentiation antigen (*CD14)* and the Toll like receptor *(TLR4)* (Additional file 2: Figure S6A) that are both known to play an important role in alcohol metabolism. We have also seen stronger enrichment of activating histone modifications at the TSS of the gene for tumor necrosis factor (*TNF4a*), which is in line with previous studies, which have shown that there is an increased expression in patients for this gene. It should also be noted the involvement of *TNF4a* in cancer which is in line with our findings on cancer described above (Additional file 2: Figure S6C). We have also observed increased enrichment of the histone modifications in the patient at the start of the genes for platelet derived growth factor (*PDGF*) and for transforming growth factor (*TGFβ1*), which are both known to mediate hepatic fibrosis, a well-known component of ASH [28] (Additional file 2: Figure S6D-E).

### Known and novel SNPs associated to disease

Single Nucleotide Polymorphisms (SNPs) are associated with many common diseases such as diabetes and high blood pressure [29]. SNPs and structural variants located in close vicinity to the TF binding motif can affect the binding of TF to DNA [30]. We identified many SNPs close to USF1 motifs in the enriched regions (Additional file 2: Table S1) and selected peaks with difference in read counts in patient and control and performed Sanger sequencing on replicate ChIP DNA to find potentially functional SNPs in these regions. No difference in genotype was found for these SNPs.

For the histone modifications we used longer paired end reads and achieved better coverage over SNPs in the differentially enriched histone regions. SNP calling on the combined dataset gave 121 SNPs in patient and 57 SNPs in control that are also present in the GWAS catalogue (http://www.genome.gov/gwastudies) (Additional file 2: Tables S2 and S3). Additionally, 1237 SNPs in high LD with the GWAS SNPs were identified in the patients and 456 in the control (Data not shown). We hypothesized that the consequence of a functional SNP sometimes can be seen as a differences in the histone modification level, due to changes in TF binding or nucleosome positioning. To support our hypothesis, we further looked at the regulatory potential of the GWAS related SNPs using two ENCODE project based databases, Haploreg [31] and RegulomeDB [32] and found that the majority of these GWAS catalogue dbSNPs are located at TF binding sites, binding motifs and DNase1 HS sites, which explains the regulatory potential of these SNPs and their possible contribution to disease. We have also observed that some of the SNPs from the patient sample are within the binding motifs of TFs associated to ASH (Additional file 2: Table S3).

Apart from GWAS catalogue SNPs, we have also identified 383 novel SNPs in the patient with overlap to the differentially modified regions (250 SNPs from regions with higher HM signal in the control and 133 SNPs from regions with higher signal in the patient) (Additional file 2: Tables S4 and S5). Some SNPs are associated to multiple genes or transcripts. Some of these SNPs might be involved in the etiology of the disease, e.g. by differential binding to TFs.

### USF1 binds genes associated to ASH

**Figure 4:**
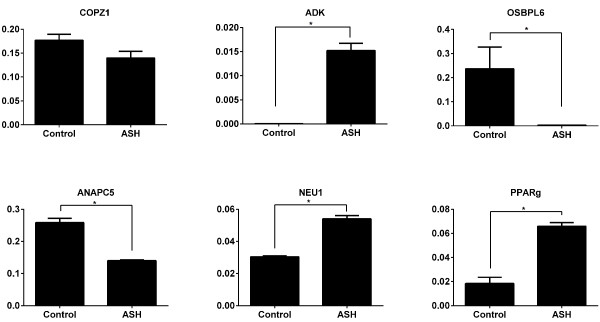
**USF1-signal and mRNA expression.** mRNA expression of the genes with differentially enriched USF1 peaks. Y-axis represents the relative expression after the normalization with GAPDH, Actin and RSP18. *P ≤ 0.05 considered as significant.

We found a good correlation between enrichment for USF1 close to TSS (r = 0.66 for peaks within 250 bp of TSS, (Additional file 2: Figure S8) with more than half of the ASH peaks located within 100 bp of a significant peak in the control dataset. Several USF1 peaks were found at genes previously known to be involved in steatohepatitis or other liver specific disorders. We compared the enrichment at these genes and found some with potential differential enrichment for USF1. We selected genes with potential differential USF1 binding where the signal was at least two times higher than the significance threshold for one of the samples, and compared the expression of genes, some with a known involvement in liver metabolism. These genes include Adenosine Kinase (*ADK*), which had a significant USF1 peak only in ASH, and Oxysterol binding protein like 6 (*OSBPL6*) and Peroxisome proliferator-activator receptor γ (*PPARγ*) [33,34], which were higher in control. ADK is an enzyme involved in liver metabolism and its deficiency may lead to the development of hepatic steatosis [34]. *PPARγ* is a TF involved in lipid metabolism and treatment with ligands of *PPARγ* can improve NASH [35]. We analyzed the mRNA expression of these genes using qRT-PCR and found the expression to agree with USF1 signal for *ADK* and *OSBPL6* but for *PPARγ* higher USF1 signal was associated with lower expression (Figure 4). We also tested three other genes not known to be involved in the disease but with potential differential USF1 binding, and found higher USF1 signals to correlate with higher expression with significant differences in mRNA for *ANAPC5* and *NEU1* but for *COPZ1* there was no significant difference in expression (Figure 4). In summary, for four of the tested genes, a significant difference in expression is seen in accordance with ChIP-seq peak heights, for one there was no difference in expression and for one a significant difference in the opposite direction was observed. USF1 may be involved in the control of expression of these genes, but differential binding does not appear to be the only mechanism underlying altered expression in ASH patients.

### ChIP-seq data of histone modifications correlated with RNA expression

**Figure 5:**
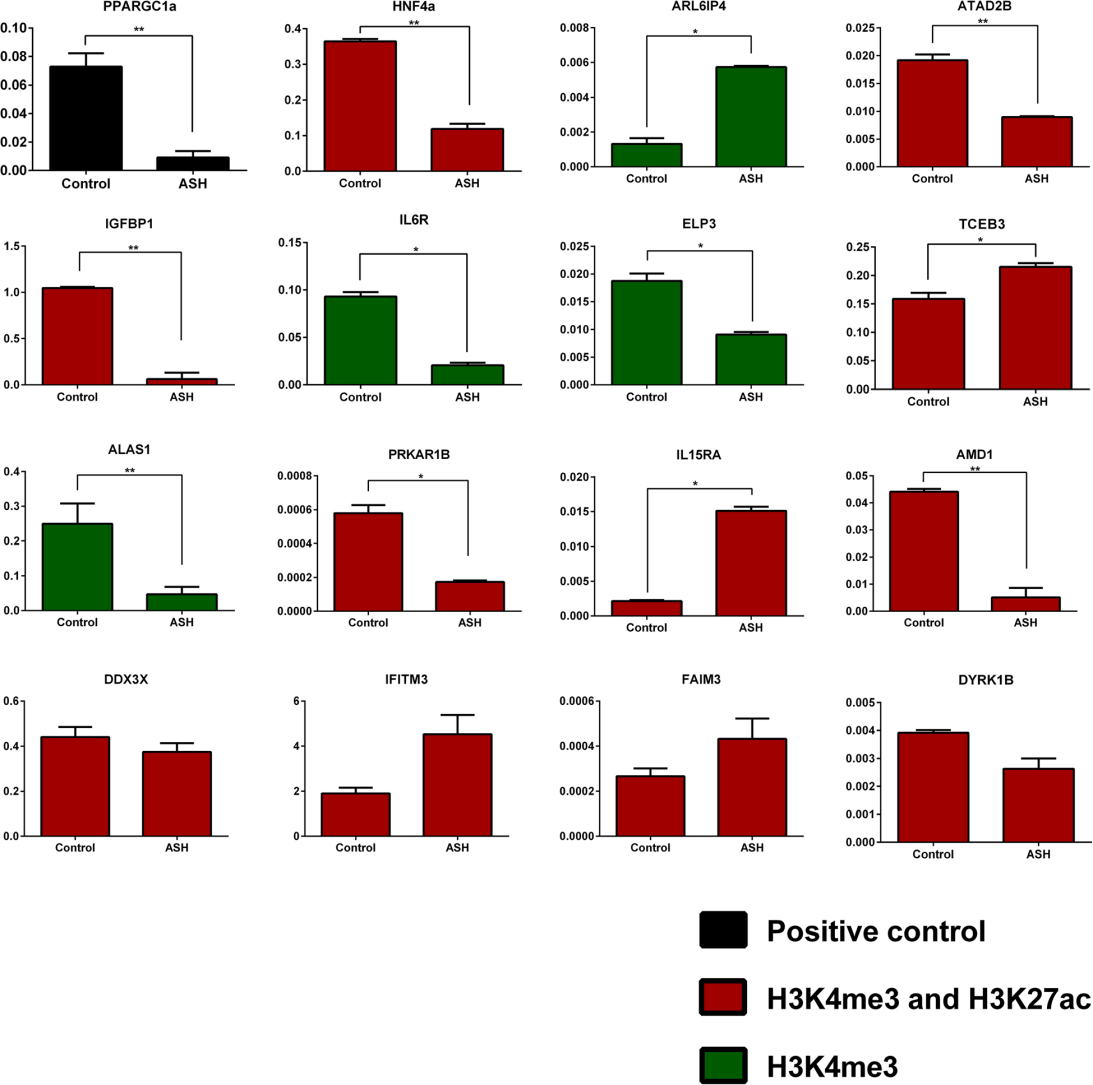
**Correlation between histone modifications and mRNA expression.** mRNA expression of the genes with differentially enriched histone modification peaks. Y-axis represents the relative expression after the normalization with GAPDH, β-Actin and RSP18. Statistically significant differences are indicated as *P < 0.05 and *P < 0.01. In green are genes with differential enrichment of H3K4me3 and in red are genes with differential enrichment of both H3K27ac and H3K4me3.

Gene expression levels can be predicted using histone modifications [36]. To see if the differential enrichment of histone modifications between ASH and controls correlate with gene expression also in other samples of the same type, we have chosen 16 genes from the top thousand differentially enriched histone modification peaks and measured RNA expression with qRT-PCR. Out of 16 genes tested with qRT-PCR (Figure 5), expression-values of 11 genes were significantly different in concordance with ChIP-seq data of histone modifications (Figure 5*; PPARGC1a, HNF4a, ARL6IP4, ATAD2B, IGFBP1, IL6R, ELP3, TCEB3, ALAS1, PRKAR1B and IL15RA*) and 1 gene had a significant change in the opposite direction (Figure 5; *AMD1*). Four genes did not show any significant difference between ASH and control (Figure 5; *DDX3X, IFITM3, FAIM3* and *DYRK1B)*. Most of the tested genes are involved in lipid metabolism and cholesterol metabolism, directly or in the presence of other TFs. We have tested Peroxisome proliferator-activated receptor γ co-activator 1α (*PPARGC1α*) which is a TF previously known to be involved in developing of NAFLD [37,38]. *PPARGC1a* gene polymorphisms and lower expression of *PPARGC1a* are important contributors of NAFLD [37] and also in our samples we found lower expression in the ASH than in the control. Thus, there is a chromatin signature related to gene expression that can help distinguish ASH from normal tissue. Further studies may define the mechanisms that drive the difference in gene activity.

## Discussion

By studying key TFs and histone modifications in tissue samples from cases with ASH and from controls we may learn more about the etiology of the disease. In a previous study, we mapped USF1 binding regions along with USF2 and H3Ac in HepG2 cell line using ChIP-chip [7] and found that USF1 regulates genes in glucose and lipid metabolism. We therefore decided to study the role of USF1 and three histone modifications in the liver disease ASH. In this study we present the first whole genome analysis of USF1 and three histone modifications using ChIP-seq in liver tissue from a patient with ASH and liver tissue with normal histology.

KEGG pathways identified many genes involved in liver metabolism in normal tissue and many cancer related genes in liver tissue from a ASH patient. This indicates that the mapping of histone modifications may provide further knowledge about disease progression. Potentially they can serve as biomarkers that could be mapped in liver biopsies from disease subjects and results like those in this study could provide support for more intense intervention to lower alcohol consumption and other types of therapy. The genes found in this study may be involved in the disease but more functional studies are needed to verify this. We have also observed the change of gene expression and function of these genes using the GORilla and Liverome databases. Based on these studies, our data proved that using ChIP-seq of histone modifications and TFs we can identify genes involved in the disease as well as new disease candidates. More functional studies of these genes are needed to determine their relation to ASH. Due to the limitation of tissue material availability from the same individual, we have used tissue material from different individuals for the ChIP-seq experiments but used the same tissues along with a third set of tissues for RNA expression and found good correlation between histone peaks and gene expression, which suggests that similar mechanisms are present in multiple patients. There was a partial correlation between USF1 ChIP-seq signal and RNA expression. The study thus gives input to the design of biobanks so that enough material is collected to allow a series of assays to be performed on a tissue sample from the same individual, or preferentially from larger cohorts.

We have identified several SNPs with high regulatory potential from the GWAS catalogue using ChIP-seq data of histone modifications. Some of these SNPs contain motifs for different TFs. For example, we found that the SNP rs17145750 is located within the binding motif of PPAR in *MLXIPL* gene (Additional file 2: Table S2). PPAR is known to be involved in NASH and ASH progression [35,37,39] and MLXIPL (also known as ChREBP) is one of the key regulators in glucose metabolism and is also involved in maintenance of tri glyceride levels [40]. Further focus on the SNPs identified in this study may reveal a contribution of these SNPs in the etiology of the disease.

From ChIP-seq data, we found many differentially enriched signals between ASH and control for USF1 and for histone modifications. RT-PCR results for mRNA expression from USF1 enriched sites showed that the expression is independent of USF1 enrichment. This means that USF1 alone did not show any effect on expression of these genes so other factors are of importance. On the other hand ChIP-seq signals for histone modifications are strongly correlated with mRNA-expression indicating that it is of interest to map them in order to learn more about the disease.

To our knowledge this is the first ChIP-seq study with USF1 and histone modifications in ASH patients. Our study identified many genes associated with liver-specific disorders. It is of great interest that we found signatures of histone modifications in genes in cancer pathways. Thus, if replicated in larger cohorts similar studies may have prognostic value in patients. We have also shown that ChIP-seq of histone modifications and TFs may aid identification of functional SNPs in regions associated to disease found in GWAS. Our study indicates the value of performing ChIP-seq with disease associated TFs to get better conclusions about the causes of disease.

## Conclusion

In conclusion we find that ChIP-seq of histone modifications and transcription factors is a powerful method to study disease mechanisms on tissue samples. Furthermore, for histone modifications we identified differentially enriched peaks i.e. that were high in disease and low in normal tissue, or vice versa, and some of the associated genes have previously been implicated in the disease whereas others should be further studied as etiological candidates. An important finding was that the highest peaks in disease tissue were at genes in cancer-specific pathways indicating that the tissue from this ASH patient could be on a route to malignancy, which is a common complication to the disease. Such histone marks have the potential to act as biomarkers for a severe disease if replicated in larger epidemiological studies. Based on GWAS, SNPs have been identified that contribute to the risk of developing steatohepatitis and other liver-specific disorders. By investigating the sequence reads of the histone modifications we found many known and novel SNPs and many of them are present in the GWAS catalogue. Since histone marks are present at gene regulatory regions the SNPs found in this study are candidates to be involved in the etiology of any disease due to molecular defects in the liver, including ASH. We have also identified genes with differentially enriched USF1 peaks and differential RNA expression which suggests that USF1 may be one of the many factors that contributes to the disease. Thus, the study shows that analysis of chromatin and TFs furthers the knowledge of liver biology and the same strategy can be applied to other normal and disease tissues.

### Data accession

All ChIP-seq data is freely available at NCBI SRA webpage (http://www.ncbi.nlm.nih.gov/sra/) with the accession number SRA066400.

## Abbreviations

ChIP: Chromatin Immunoprecipitation; ChIP-seq: Chromatin Immunoprecipitation coupled with sequencing; GO: Gene ontology; GWAS: Genome wide association studies; H3K27ac: Histone 3 lysine 27 acetylation; H3K4me1: Histone 3 lysine 4 mono-methylation; H3K4me3: Histone 3 lysine 4 tri-methylation; HCC: Hepatocellular carcinoma; NAFLD: Non-alcoholic fatty liver disease; ASH: Alcoholic steatohepatitis; NASH: Non-alcoholic steatohepatitis; qRT-PCR: Quantitative real time PCR; SNP: Single nucleotide polymorphism; TF: Transcription factor; TSS: Transcription start site; USF1: Upstream stimulatory factor 1.

## Competing interests

The authors declare that they have no competing interests.

## Authors’ contributions

CW and KZ conceived the study and CW coordinated experiments and analysis. MSB performed most of the experiments. MSB, OW and SB analyzed data after input from CW and JK. MSB wrote a draft of the manuscript and major editing was done by CW, OW and SB. All authors read and approved the final version of the manuscript.

## Pre-publication history

The pre-publication history for this paper can be accessed here:

http://www.biomedcentral.com/1755-8794/6/50/prepub

## Supplementary Material

Additional file 1 Shows the USF1 peaks and read counts for control and ASH. Click here for file

Additional file 2 Figure S1 Comparison of ChIP-seq signals with ChIP-qPCR represents the good correlation between qPCR and ChIP-seq signal. **Figure S2.** Comparison of the ChIP-seq signal over the peak regions between disease and control for the histone modifications. **Figure S3.** The number of genes that contain histone modification peaks both in ASH and in control, only in ASH and only in control. **Figure S4.** Different biological processes identified using the genes associated with histone modifications in ASH. **Figure S5.** Different biological processes identified using the genes associated with histone modifications in control. **Figure S6.** Histone modification pattern for the genes associated with alcoholic liver disease and ASH. **Figure S7.** Fraction of peaks with USF1 motif ranked on peak height, with comparison to peaks called with MACS for the same dataset. **Figure S8.** Correlation between ASH and control signals for USF1 for peaks close to TSS. **Table S1.** Sanger sequencing results of SNPs identified at USF1 peaks and alleles identified for Genomic DNA and ChIP DNA of USF1. Allele frequencies obtained from dbSNP129 and AA, AB and BB indicate the frequencies calculated by using *Hardy-Weinberg equation*. **Table S2.** GWAS catalogue dbSNPs identified using ChIP-seq data of histone modifications in control. **Table S3.** GWAS catalogue dbSNPs identified using ChIP-seq data of histone modifications in ASH. **Table S4.** Novel SNPs identified using ChIP-seq data of histone modifications in control. **Table S5.** Novel SNPs identified using ChIP-seq data of histone modifications in ASH. **Table S6.** Primers used for USF1 qPCR validations, mRNA primers for USF1 and histone modifications. Click here for file

Additional file 3 Peak regions with the highest differences in histone modification levels between ASH and control. Click here for file

Additional file 4 **List of genes within 2 kb from the histone modification peaks with highest difference between ASH and control.** Peaks for all three histone modifications are combined. This is the list we have used for Gene Ontology. Click here for file
