# Supplementary material for: ChIP-seq in steatohepatitis and normal liver tissue identifies candidate disease mechanisms related to progression to cancer
Source: BMC Med Genomics. 2013 Nov 8;6:50. doi: 10.1186/1755-8794-6-50 (PMC3831757; doi:10.1186/1755-8794-6-50)
Supplement: Additional file 2 Figure S1 — Comparison of ChIP-seq signals with ChIP-qPCR represents the good correlation between qPCR and ChIP-seq signal. Figure S2. Comparison of the ChIP-seq signal over the peak regions between disease and control for the histone modifications. Figure S3. The number of genes that contain histone modification peaks both in ASH and in control, only in ASH and only in control. Figure S4. Different biological processes identified using the genes associated with histone modifications in ASH. Figure S5. Different biological processes identified using the genes associated with histone modifications in control. Figure S6. Histone modification pattern for the genes associated with alcoholic liver disease and ASH. Figure S7. Fraction of peaks with USF1 motif ranked on peak height, with comparison to peaks called with MACS for the same dataset. Figure S8. Correlation between ASH and control signals for USF1 for peaks close to TSS. Table S1. Sanger sequencing results of SNPs identified at USF1 peaks and alleles identified for Genomic DNA and ChIP DNA of USF1. Allele frequencies obtained from dbSNP129 and AA, AB and BB indicate the frequencies calculated by using Hardy-Weinberg equation. Table S2. GWAS catalogue dbSNPs identified using ChIP-seq data of histone modifications in control. Table S3. GWAS catalogue dbSNPs identified using ChIP-seq data of histone modifications in ASH. Table S4. Novel SNPs identified using ChIP-seq data of histone modifications in control. Table S5. Novel SNPs identified using ChIP-seq data of histone modifications in ASH. Table S6. Primers used for USF1 qPCR validations, mRNA primers for USF1 and histone modifications. [file 1755-8794-6-50-S2.docx]

**ChIP-seq in steatohepatitis and normal liver tissue identifies candidate disease mechanisms related to progression to cancer**

Madhusudhan Reddy Bysani^1,2^, Ola Wallerman^1,2,6^, Susanne Bornelöv^1,3^,, Kurt Zatloukal^3^, Jan Komorowski^1,3,5^, Claes Wadelius^1,2 *^.

1. Science for Life Laboratory, Uppsala University, Uppsala, Sweden.
2. Department of Immunology, Genetics and Pathology, Rudbeck Laboratory, Uppsala University, Uppsala, Sweden.
3. Department of Cell and Molecular Biology, BMC, Uppsala.
4. Institute of Pathology, Medical University of Graz, Austria.
5. Interdisciplinary Centre for Mathematical and Computational Modelling, University of Warsaw, PL-02-106 Warszawa, Poland.
6. Current address: Department of Medical Biochemistry and Microbiology, BMC, Uppsala.

^*^Corresponding author: E mail: Claes.Wadelius@igp.uu.se, Fax: +46-18-471-4808.

**Additional Figures and Tables**

Figure S1: Comparison of ChIP-seq signals with ChIP-qPCR represents the good correlation between qPCR and ChIP-seq signal.

Figure S2: Comparison of the ChIP-seq signal over the peak regions between disease and control for the histone modifications.

Figure S3: The number of genes that contain histone modification peaks both in ASH and in control, only in ASH and only in control.

Figure S4: Different biological processes identified using the genes associated with histone modifications in ASH.

Figure S5: Different biological processes identified using the genes associated with histone modifications in control.

Figure S6: Histone modification pattern for the genes associated with alcoholic liver disease and ASH.

Table S1: Sanger sequencing results of SNPs identified at USF1 peaks and alleles identified for Genomic DNA and ChIP DNA of USF1. Allele frequencies obtained from dbSNP129 and AA, AB and BB indicate the frequencies calculated by using *Hardy-Weinberg equation*.

Table S2: GWAS catalogue dbSNPs identified using ChIP-seq data of histone modifications in control.

Table S3: GWAS catalogue dbSNPs identified using ChIP-seq data of histone modifications in ASH.

Table S4: Novel SNPs identified using ChIP-seq data of histone modifications in control.

Table S5: Novel SNPs identified using ChIP-seq data of histone modifications in ASH.

Table S6: Primers used for USF1 qPCR validations, mRNA primers for USF1 and histone modifications.

Figure S1: Comparison of ChIP-seq signals with ChIP-qPCR.

Figure S2: Comparison of the ChIP-seq signal over the peak regions.

**H3K4me1**


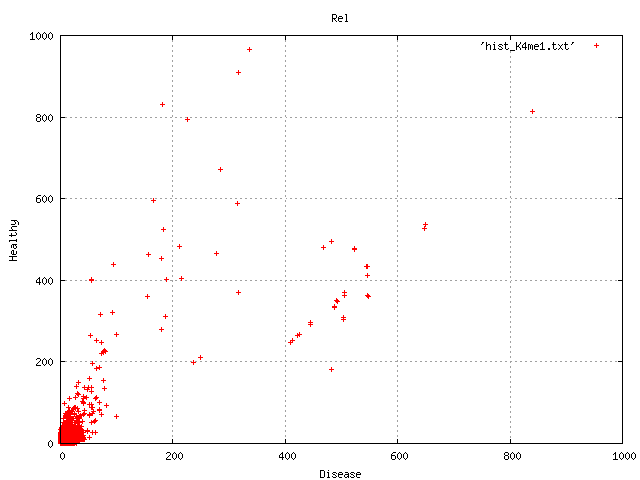


**H3K4me3**

**
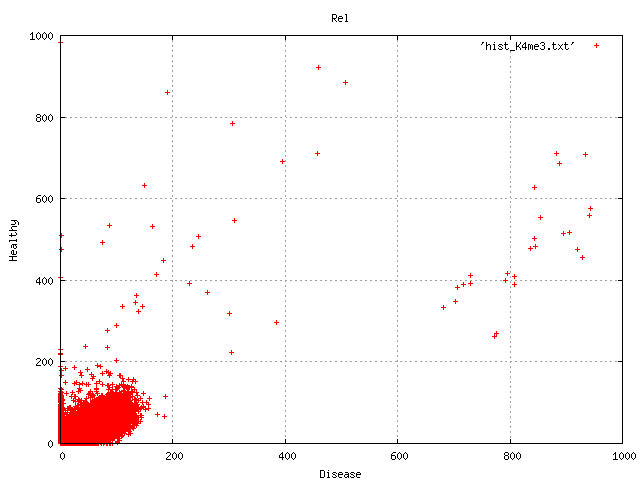
**

**H3K27ac**


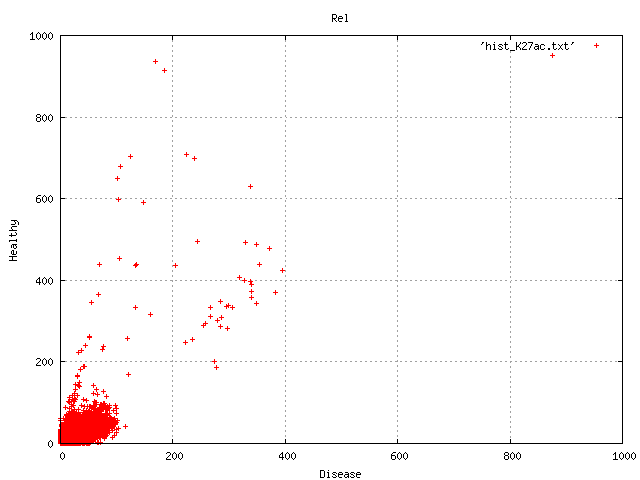


Figure S3: Number of genes that contain histone modification peaks.

Figure S4: Biological processes in ASH compared to control.


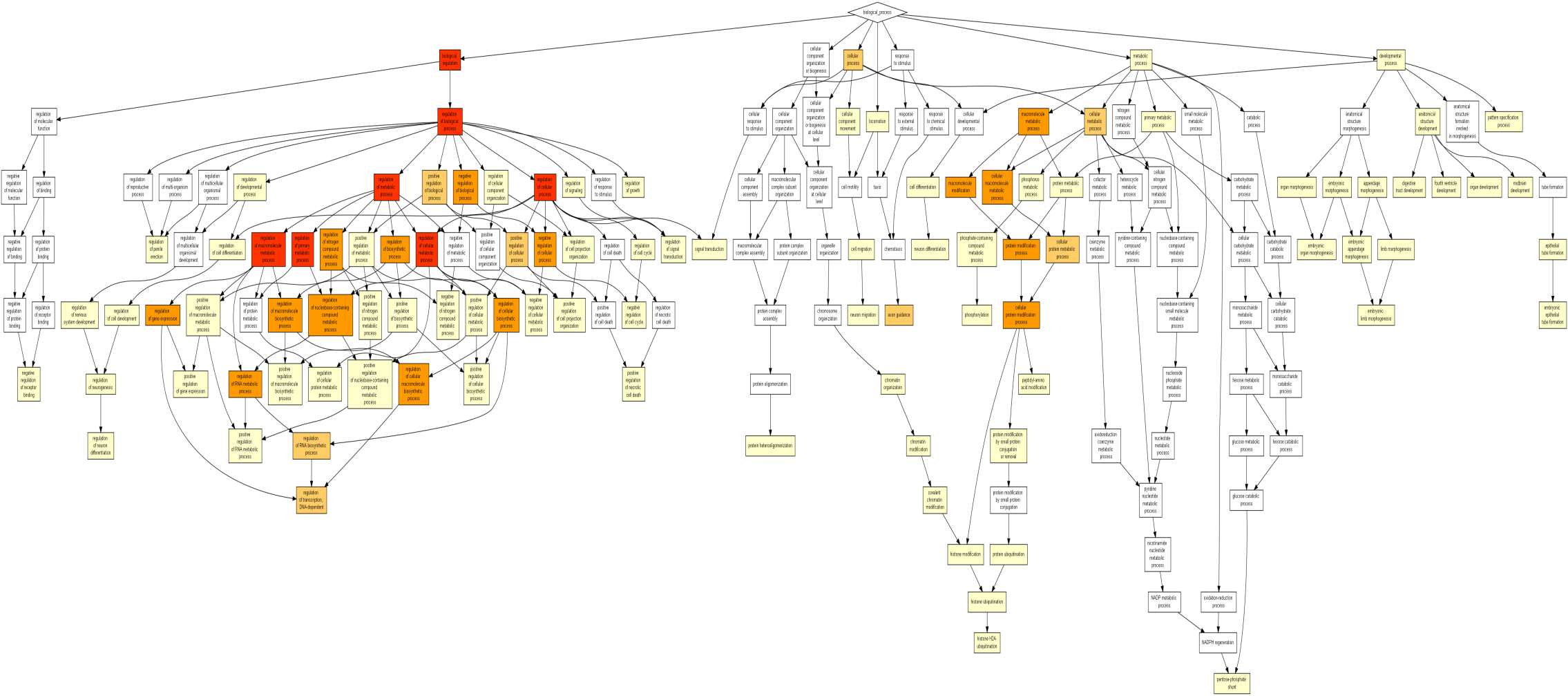


Figure S5: Biological processes in control compared to ASH.


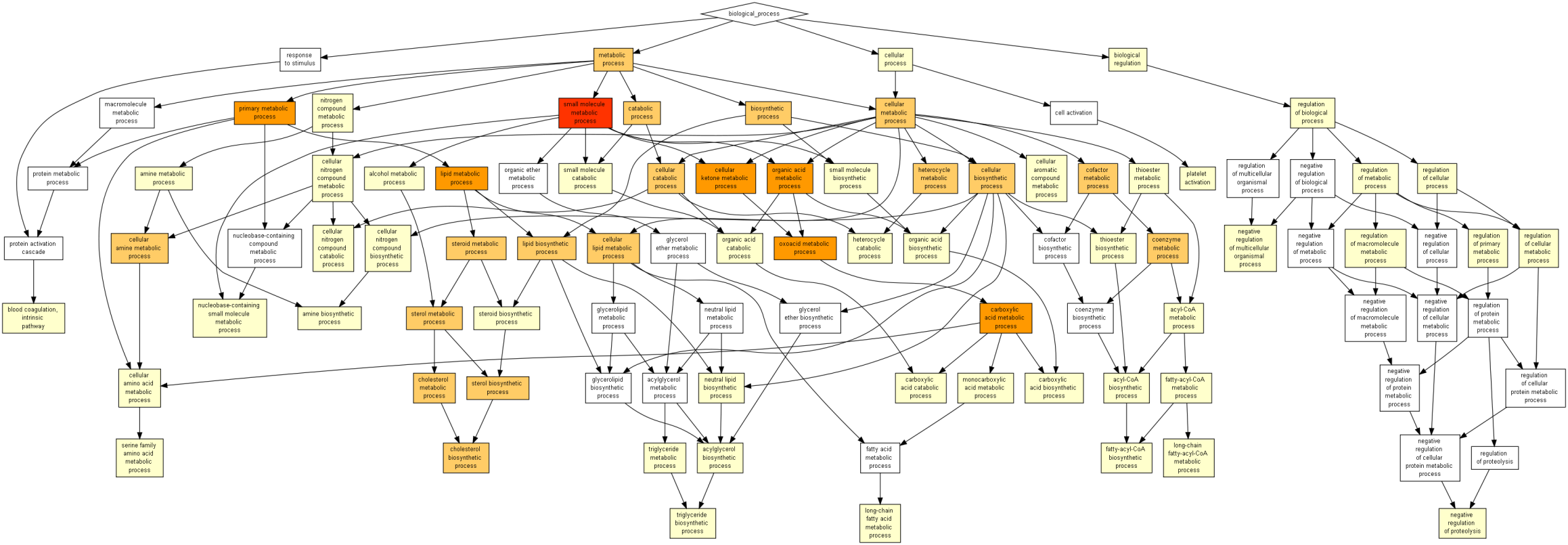


Figure S6: Histone modification patterns observed for the genes associated with Alcoholic liver disease.


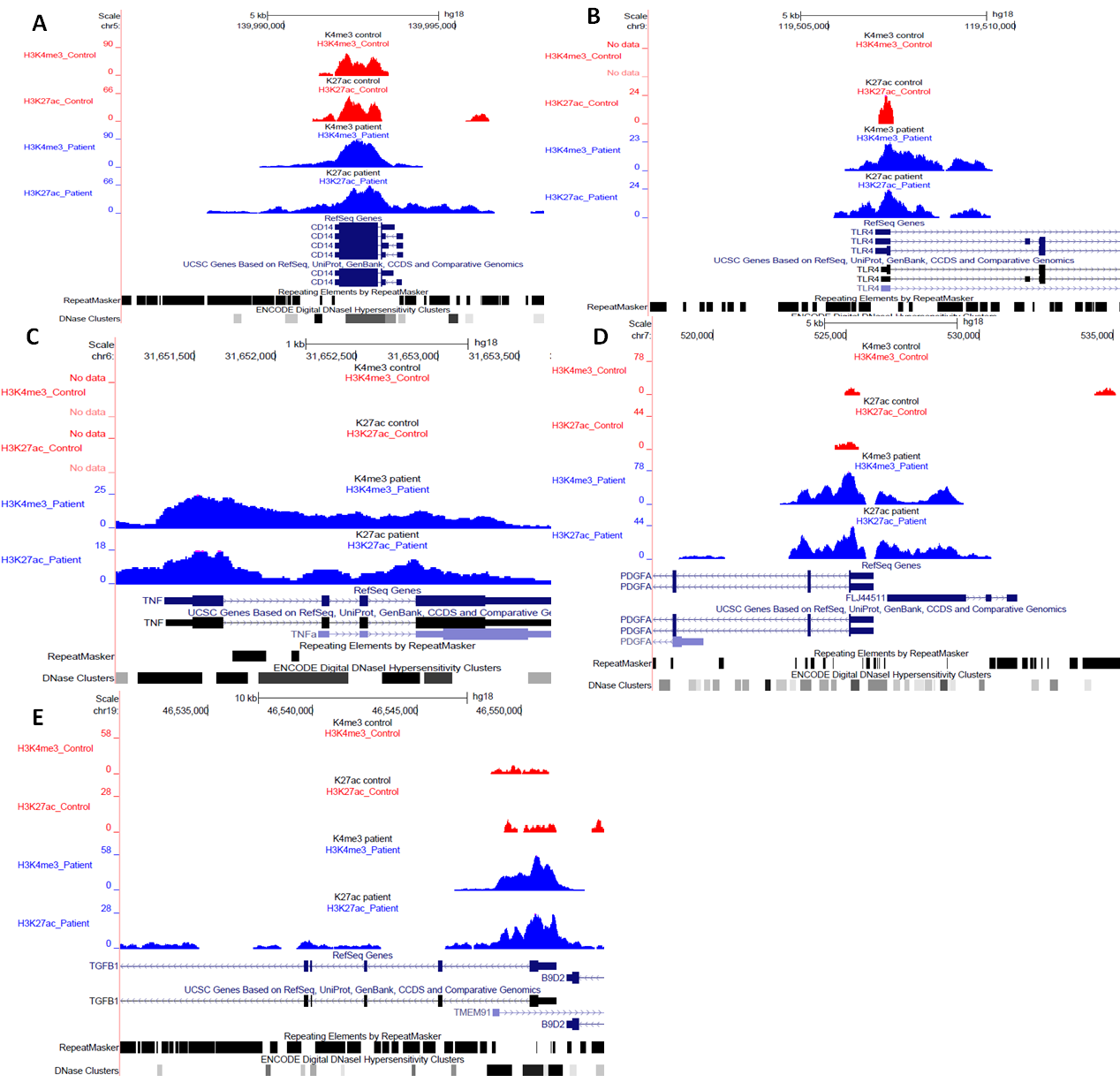


**Figure S7.** Enrichment of CACGTG sequences at peak centers for peaks with different enrichment. Peaks were ranked by peak height and the fraction of peaks with a match to CACGTG within 50 bp of the summit was calculated for each bin with 100 peaks. Blu line shows the peak list used in the manuscript, red shows the MACS peak calls (summits) on the same data.

**Figure S8**. Correlation between USF1 enrichment in ASH and control for peaks within 250 bp of TSS.


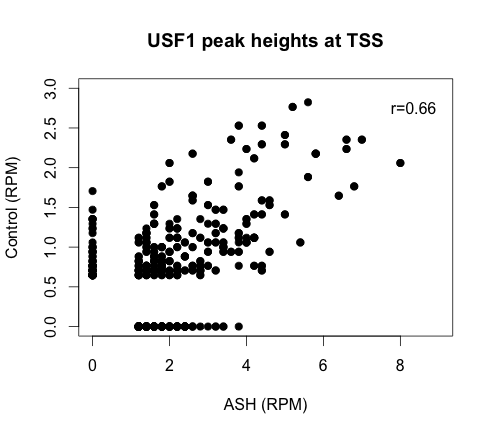


Table S1: Sanger sequencing results of SNPs identified at USF1 peaks.

| SNP ID | Genomic ASH | Genomic Control | ChIP ASH | ChIP Control | Allele Freq | AA | AB | BB |  |
| --- | --- | --- | --- | --- | --- | --- | --- | --- | --- |
| rs35239455 | G | G | G | G |  |  |  |  |  |
| rs56258811 | C | C | C | C |  |  |  |  |  |
| rs7939948 | T | T | T | T | 0,47 (G); 0.53 (T) | 0,22 | 0,498 | 0,28 |  |
| rs4645910 | A | A | A | A | 0,94 (A); 0,06 (C) | 0,883 | 0,112 | 0,0036 |  |
| rs34110942 | G | G | G | G |  |  |  |  |  |
| rs941677 | C | C | C | C | 0,1 (T); 0,9 (C) | 0,01 | 0,18 | 0,81 |  |
| rs3810000 | G | G | G | G |  |  |  |  |  |
| rs28372781 | G | G | G | G | 0,9737(G); 0,0263 (C) | 0,948092 | 0,051217 | 0,000692 |  |
| rs16980380 |  |  |  |  | 0,9787 (T); 0,0212(C) | 0,957854 | 0,041497 | 0,000449 |  |
| rs55738654 | A | A | A | A |  |  |  |  |  |
| rs4457792 | G | G | G | G | 0,8649 (G); 0,135 (C) | 0,748052 | 0,233523 | 0,018225 |  |
| rs1322815 | T | T | T | T |  |  |  |  |  |
| rs17132319 | T | T | T | T | 0,0608(G); 0,9391 (T) | 0,003697 | 0,114195 | 0,881909 |  |

Table S2: SNPs identified in control also present in the GAWS catalogue.

| **chr** | **pos (hg18)** | **LD** | **Variant** | **Ref** | **Alt** | **DNAse** |  |  |  |  |  |
| --- | --- | --- | --- | --- | --- | --- | --- | --- | --- | --- | --- |
|  |  |  |  |  |  |  | **Proteins bound** | **Motifs Changed** | **GENCODE genes** | **Ref seq Genes** | **Motifs from Regulome DB** |
| 1 | 114179091 | 1 | [rs2476601](http://www.broadinstitute.org/mammals/haploreg/detail.php?query=&id=rs2476601) | A | G | 5 cell types | STAT3 | GABP | PTPN22 | PTPN22 | PU.1, GABPA |
| 1 | 232925220 | 1 | [rs514230](http://www.broadinstitute.org/mammals/haploreg/detail.php?query=&id=rs514230) | A | T | HA-h,Jurkat |  | EWSR1-FLI1 | 2.6kb 5' of RP4-781K5.8 | 1.2kb 5' of LOC100506810 | CAC-binding protein |
| 1 | 16377907 | 1 | [rs1497406](http://www.broadinstitute.org/mammals/haploreg/detail.php?query=&id=rs1497406) | A | G | 16 cell types | 12 bound proteins | Otx2,Gsc,Pitx2 | 17kb 3' of RP11-276H7.3 | 19kb 3' of ARHGEF19 | Otx2,Gsc,Pitx2 |
| 1 | 109619361 | 1 | [rs660240](http://www.broadinstitute.org/mammals/haploreg/detail.php?query=&id=rs660240) | T | C | 25 cell types | PU1,TCF4,CJUN |  | CELSR2 | CELSR2 |  |
| 1 | 228362314 | 1 | [rs4846914](http://www.broadinstitute.org/mammals/haploreg/detail.php?query=&id=rs4846914) | G | A | Hepatocytes,LNCaP |  |  | GALNT2 | GALNT2 |  |
| 1 | 21694344 | 1 | [rs1780324](http://www.broadinstitute.org/mammals/haploreg/detail.php?query=&id=rs1780324) | A | G | K562,pHTE |  |  | 10kb 3' of NBPF3 | 10kb 5' of NBPF3 |  |
| 2 | 43918594 | 1 | [rs6756629](http://www.broadinstitute.org/mammals/haploreg/detail.php?query=&id=rs6756629) | G | A |  |  |  | ABCG5 | ABCG5 |  |
| 2 | 169471394 | 1 | [rs560887](http://www.broadinstitute.org/mammals/haploreg/detail.php?query=&id=rs560887) | T | C |  |  |  | G6PC2 | G6PC2 |  |
| 2 | 21117405 | 1 | [rs1367117](http://www.broadinstitute.org/mammals/haploreg/detail.php?query=&id=rs1367117) | G | A |  | HEY1,POL2 |  | APOB | APOB |  |
| 2 | 43926080 | 1 | [rs4299376](http://www.broadinstitute.org/mammals/haploreg/detail.php?query=&id=rs4299376) | G | T |  |  |  | ABCG8 | ABCG8 |  |
| 2 | 233221419 | 1 | [rs2140773](http://www.broadinstitute.org/mammals/haploreg/detail.php?query=&id=rs2140773) | C | A | 10 cell types |  | RREB-1 | EFHD1 | EFHD1 |  |
| 2 | 43927385 | 1 | [rs6544713](http://www.broadinstitute.org/mammals/haploreg/detail.php?query=&id=rs6544713) | T | C |  |  |  | ABCG8 | ABCG8 |  |
| 2 | 160879700 | 1 | [rs7593730](http://www.broadinstitute.org/mammals/haploreg/detail.php?query=&id=rs7593730) | T | C |  |  |  | RBMS1 | RBMS1 |  |
| 2 | 27596658 | 1 | [rs780092](http://www.broadinstitute.org/mammals/haploreg/detail.php?query=&id=rs780092) | A | G |  |  |  | GCKR | GCKR | Klf4 |
| 4 | 6353923 | 1 | [rs1801214](http://www.broadinstitute.org/mammals/haploreg/detail.php?query=&id=rs1801214) | C | T | 7 cell types |  |  | WFS1 | WFS1 |  |
| 5 | 74691482 | 1 | [rs3846663](http://www.broadinstitute.org/mammals/haploreg/detail.php?query=&id=rs3846663) | C | T |  |  |  | HMGCR | HMGCR |  |
| 5 | 74684359 | 1 | [rs12654264](http://www.broadinstitute.org/mammals/haploreg/detail.php?query=&id=rs12654264) | A | T |  |  |  | HMGCR | HMGCR |  |
| 6 | 93967840 | 1 | [rs12527818](http://www.broadinstitute.org/mammals/haploreg/detail.php?query=&id=rs12527818) | T | C |  |  |  | 39kb 3' of EPHA7 | 39kb 3' of EPHA7 |  |
| 7 | 47746494 | 1 | [rs7800244](http://www.broadinstitute.org/mammals/haploreg/detail.php?query=&id=rs7800244) | G | T | Huh-7.5 |  | Pax-3,Pax-4 | HUS1 | 21kb 5' of MGC16075 |  |
| 7 | 72620810 | 1 | [rs17145738](http://www.broadinstitute.org/mammals/haploreg/detail.php?query=&id=rs17145738) | C | T | 5 cell types |  | Msx-1 | 1.1kb 3' of TBL2 | 401bp 3' of TBL2 | Msx-1 |
| 8 | 126562928 | 1 | [rs2954033](http://www.broadinstitute.org/mammals/haploreg/detail.php?query=&id=rs2954033) | A | G | HTR8svn |  |  | RP11-136O12.2 | 43kb 5' of TRIB1 |  |
| 8 | 9215678 | 1 | [rs6984305](http://www.broadinstitute.org/mammals/haploreg/detail.php?query=&id=rs6984305) | A | T | 45 cell types | 4 bound proteins | CTCF | RP11-115J16.1 | 4.3kb 5' of LOC157273 |  |
| 8 | 9222556 | 1 | [rs2126259](http://www.broadinstitute.org/mammals/haploreg/detail.php?query=&id=rs2126259) | T | C |  |  |  | RP11-115J16.1 | LOC157273 |  |
| 8 | 126547160 | 1 | [rs2001945](http://www.broadinstitute.org/mammals/haploreg/detail.php?query=&id=rs2001945) | G | C | 4 cell types |  |  | 1.2kb 5' of RP11-136O12.2 | 27kb 5' of TRIB1 |  |
| 8 | 96029687 | 1 | [rs896854](http://www.broadinstitute.org/mammals/haploreg/detail.php?query=&id=rs896854) | T | C | 9 cell types |  |  | C8orf38 | TP53INP1 |  |
| 8 | 9221006 | 1 | [rs4841132](http://www.broadinstitute.org/mammals/haploreg/detail.php?query=&id=rs4841132) | A | G |  |  |  | RP11-115J16.1 | LOC157273 |  |
| 8 | 9220768 | 1 | [rs9987289](http://www.broadinstitute.org/mammals/haploreg/detail.php?query=&id=rs9987289) | A | G |  |  |  | RP11-115J16.1 | LOC157273 |  |
| 9 | 4277466 | 1 | [rs7041847](http://www.broadinstitute.org/mammals/haploreg/detail.php?query=&id=rs7041847) | A | G | HRGEC,LNCaP |  |  | GLIS3 | GLIS3 |  |
| 9 | 22019547 | 1 | [rs564398](http://www.broadinstitute.org/mammals/haploreg/detail.php?query=&id=rs564398) | T | C |  |  |  | MTAP | CDKN2B-AS1 | Osf2 |
| 10 | 94337810 | 1 | [rs6583826](http://www.broadinstitute.org/mammals/haploreg/detail.php?query=&id=rs6583826) | G | A |  |  |  | 5.2kb 5' of KIF11 | 5kb 5' of KIF11 | Sox15 |
| 10 | 101785351 | 1 | [rs10883437](http://www.broadinstitute.org/mammals/haploreg/detail.php?query=&id=rs10883437) | T | A |  |  | PLZF | 6.6kb 3' of CPN1 | 6.7kb 3' of CPN1 |  |
| 10 | 94829632 | 1 | [rs2068888](http://www.broadinstitute.org/mammals/haploreg/detail.php?query=&id=rs2068888) | G | A | 8 cell types | CEBPB | GATA | 2kb 3' of CYP26A1 | 2kb 5' of CYP26A1 | GATA |
| 11 | 116112647 | 1 | [rs1558861](http://www.broadinstitute.org/mammals/haploreg/detail.php?query=&id=rs1558861) | C | T | 8988T |  |  | 11kb 3' of BUD13 | 11kb 3' of BUD13 |  |
| 11 | 65106332 | 1 | [rs6591182](http://www.broadinstitute.org/mammals/haploreg/detail.php?query=&id=rs6591182) | T | G | 12 cell types |  | OLF1, PPAR,SZF-1, MYF-4 | EHBP1L1 | EHBP1L1 | PPARa-RXRa |
| 11 | 2138800 | 1 | [rs689](http://www.broadinstitute.org/mammals/haploreg/detail.php?query=&id=rs689) | A | T | 15 cell types |  | Ik-1 | INS | INS | LYF1 |
| 11 | 92331180 | 1 | [rs2166706](http://www.broadinstitute.org/mammals/haploreg/detail.php?query=&id=rs2166706) | T | C |  |  |  | 7.4kb 5' of RP11-676F20.1 | 11kb 5' of MTNR1B |  |
| 11 | 116167789 | 1 | [rs651821](http://www.broadinstitute.org/mammals/haploreg/detail.php?query=&id=rs651821) | C | T | 5 cell types |  |  | APOA5 | APOA5 |  |
| 11 | 61379716 | 1 | [rs174601](http://www.broadinstitute.org/mammals/haploreg/detail.php?query=&id=rs174601) | C | T |  |  |  | FADS1 | FADS2 |  |
| 11 | 2648047 | 1 | [rs231362](http://www.broadinstitute.org/mammals/haploreg/detail.php?query=&id=rs231362) | A | G |  |  | Stra13 | KCNQ1 | KCNQ1OT1 |  |
| 11 | 116165896 | 1 | [rs2266788](http://www.broadinstitute.org/mammals/haploreg/detail.php?query=&id=rs2266788) | G | A | 8 cell types |  |  | APOA5 | APOA5 |  |
| 11 | 116237722 | 1 | [rs2075292](http://www.broadinstitute.org/mammals/haploreg/detail.php?query=&id=rs2075292) | G | T | 58 cell types | 5 bound proteins |  | SIK3 | SIK3 |  |
| 11 | 116154127 | 1 | [rs964184](http://www.broadinstitute.org/mammals/haploreg/detail.php?query=&id=rs964184) | G | C | HAEpiC,HVMF |  | Foxj2 | ZNF259 | 357bp 3' of ZNF259 | FOXJ2 |
| 12 | 101436688 | 1 | [rs35747](http://www.broadinstitute.org/mammals/haploreg/detail.php?query=&id=rs35747) | G | A |  |  | Pbx | 38kb 5' of IGF1 | 38kb 3' of | PBX |
| 12 | 110368991 | 1 | [rs3184504](http://www.broadinstitute.org/mammals/haploreg/detail.php?query=&id=rs3184504) | T | C | NHEK,WI-38 |  | HES1,Mtf1 | SH2B3 | SH2B3 | Mtf1 |
| 14 | 100212643 | 1 | [rs730570](http://www.broadinstitute.org/mammals/haploreg/detail.php?query=&id=rs730570) | G | A | 23 cell types |  |  | 3.8kb 3' of C14orf70 | 3.8kb 5' of C14orf70 |  |
| 14 | 22909873 | 1 | [rs10137082](http://www.broadinstitute.org/mammals/haploreg/detail.php?query=&id=rs10137082) | C | T | HeLa-S3,Huh-7.5 |  |  | 2kb 5' of IL25 | 2kb 5' of IL25 |  |
| 15 | 56470658 | 1 | [rs1532085](http://www.broadinstitute.org/mammals/haploreg/detail.php?query=&id=rs1532085) | A | G |  |  |  | 19kb 5' of LIPC | 41kb 5' of LIPC |  |
| 16 | 52366748 | 1 | [rs1121980](http://www.broadinstitute.org/mammals/haploreg/detail.php?query=&id=rs1121980) | G | A | NB4 | PU1,USF1 |  | FTO | FTO |  |
| 17 | 2209453 | 1 | [rs4790333](http://www.broadinstitute.org/mammals/haploreg/detail.php?query=&id=rs4790333) | C | T | 66 cell types | 6 bound proteins |  | SGSM2 | SGSM2 |  |
| 19 | 50106291 | 1 | [rs439401](http://www.broadinstitute.org/mammals/haploreg/detail.php?query=&id=rs439401) | T | C | 46 cell types | 7 bound proteins | UF1H3BETA,Sp1,VDR | 1.8kb 3' of APOE | 1.8kb 5' of APOE | VDR |
| 19 | 50087106 | 1 | [rs157580](http://www.broadinstitute.org/mammals/haploreg/detail.php?query=&id=rs157580) | G | A | 9 cell types |  |  | TOMM40 | TOMM40 |  |
| 20 | 1558551 | 1 | [rs2281808](http://www.broadinstitute.org/mammals/haploreg/detail.php?query=&id=rs2281808) | T | C |  |  |  | SIRPG | SIRPG |  |
| 20 | 25246087 | 1 | [rs7267979](http://www.broadinstitute.org/mammals/haploreg/detail.php?query=&id=rs7267979) | A | G |  |  |  | ABHD12 | ABHD12 |  |
| 21 | 43592990 | 1 | [rs643608](http://www.broadinstitute.org/mammals/haploreg/detail.php?query=&id=rs643608) | C | T | 7 cell types |  | Ets | 9.5kb 3' of AP001046.5 | 66kb 3' of SIK1 |  |
| 22 | 35921264 | 1 | [rs229541](http://www.broadinstitute.org/mammals/haploreg/detail.php?query=&id=rs229541) | G | A |  |  | GATA | C1QTNF6 | 7kb 3' of C1QTNF6 | GATA5 |
| X | 152553116 | 1 | [rs5945326](http://www.broadinstitute.org/mammals/haploreg/detail.php?query=&id=rs5945326) | A | G | 10 cell types |  |  | 8kb 5' of DUSP9 | 8kb 5' of DUSP9 |  |

Table S3: SNPs identified in ASH also present in the GAWS catalogue.

| **chr** | **pos (hg18)** | **LD** | **Variant** | **Ref** | **Alt** | **DNAse** |  |  |  |  |
| --- | --- | --- | --- | --- | --- | --- | --- | --- | --- | --- |
|  |  |  |  |  |  |  | **Proteins bound** | **Motifs changed** | **GENCODE genes** | **Motifs from Regulome DB** |
| 1 | 205006527 | 1 | [rs3024505](http://www.broadinstitute.org/mammals/haploreg/detail.php?query=&id=rs3024505) | G | A | 74 cell types | 22 bound proteins |  | 1kb 3' of IL10 | MZF-1 |
| 1 | 205941798 | 1 | [rs4844614](http://www.broadinstitute.org/mammals/haploreg/detail.php?query=&id=rs4844614) | G | T |  |  |  | CR1L |  |
| 1 | 55277238 | 1 | [rs2479409](http://www.broadinstitute.org/mammals/haploreg/detail.php?query=&id=rs2479409) | G | A | WI-38 |  | Mrg1,Hoxa9,ATF | 570bp 5' of PCSK9 | ATF |
| 1 | 16377907 | 1 | [rs1497406](http://www.broadinstitute.org/mammals/haploreg/detail.php?query=&id=rs1497406) | A | G | 16 cell types | 12 bound proteins | Otx2,Gsc,Pitx2 | 17kb 3' of RP11-276H7.3 | Otx2,Gsc,Pitx2, DOBOX5 |
| 1 | 21639040 | 1 | [rs1976403](http://www.broadinstitute.org/mammals/haploreg/detail.php?query=&id=rs1976403) | A | C | 48 cell types | SIN3AK20,TCF4,POL2 |  | 167bp 5' of NBPF3 |  |
| 1 | 95464898 | 1 | [rs1414896](http://www.broadinstitute.org/mammals/haploreg/detail.php?query=&id=rs1414896) | G | A |  |  |  | RP11-57H12.3 |  |
| 1 | 228362314 | 1 | [rs4846914](http://www.broadinstitute.org/mammals/haploreg/detail.php?query=&id=rs4846914) | G | A | Hepatocytes,LNCaP |  |  | GALNT2 |  |
| 1 | 62769426 | 1 | [rs1168013](http://www.broadinstitute.org/mammals/haploreg/detail.php?query=&id=rs1168013) | C | G |  |  | NF-I,Barx1 | DOCK7 |  |
| 1 | 62704220 | 1 | [rs1167998](http://www.broadinstitute.org/mammals/haploreg/detail.php?query=&id=rs1167998) | C | A |  |  | Hoxc9 | DOCK7 |  |
| 1 | 228361539 | 1 | [rs2144300](http://www.broadinstitute.org/mammals/haploreg/detail.php?query=&id=rs2144300) | C | T | 9 cell types | 5 bound proteins |  | GALNT2 |  |
| 2 | 24546313 | 1 | [rs2165738](http://www.broadinstitute.org/mammals/haploreg/detail.php?query=&id=rs2165738) | C | G | SK-N-MC |  | GCNF | 22kb 5' of NCOA1 |  |
| 2 | 160879700 | 1 | [rs7593730](http://www.broadinstitute.org/mammals/haploreg/detail.php?query=&id=rs7593730) | T | C |  |  |  | RBMS1 |  |
| 2 | 112487241 | 1 | [rs4374383](http://www.broadinstitute.org/mammals/haploreg/detail.php?query=&id=rs4374383) | A | G |  |  |  | MERTK |  |
| 2 | 27594741 | 1 | [rs780094](http://www.broadinstitute.org/mammals/haploreg/detail.php?query=&id=rs780094) | T | C | HepG2 | FOXA2,RXRA,MAFK | MafB | GCKR | MAF-b |
| 2 | 21139562 | 1 | [rs515135](http://www.broadinstitute.org/mammals/haploreg/detail.php?query=&id=rs515135) | T | C |  |  |  | 19kb 5' of APOB |  |
| 2 | 43927385 | 1 | [rs6544713](http://www.broadinstitute.org/mammals/haploreg/detail.php?query=&id=rs6544713) | T | C |  |  |  | ABCG8 |  |
| 2 | 21117405 | 1 | [rs1367117](http://www.broadinstitute.org/mammals/haploreg/detail.php?query=&id=rs1367117) | G | A |  | HEY1,POL2 |  | APOB |  |
| 2 | 43926080 | 1 | [rs4299376](http://www.broadinstitute.org/mammals/haploreg/detail.php?query=&id=rs4299376) | G | T |  |  |  | ABCG8 |  |
| 2 | 27584444 | 1 | [rs1260326](http://www.broadinstitute.org/mammals/haploreg/detail.php?query=&id=rs1260326) | T | C |  |  | NRSF | GCKR |  |
| 2 | 9262599 | 1 | [rs6487679](http://www.broadinstitute.org/mammals/haploreg/detail.php?query=&id=rs6487679) | C | T |  |  | Foxl1 | 9.1kb 3' of U7.41 | CEBP |
| 2 | 112487241 | 1 | [rs4374383](http://www.broadinstitute.org/mammals/haploreg/detail.php?query=&id=rs4374383) | A | G |  |  |  | MERTK |  |
| 2 | 233221419 | 1 | [rs2140773](http://www.broadinstitute.org/mammals/haploreg/detail.php?query=&id=rs2140773) | C | A | 10 cell types |  | RREB-1 | EFHD1 |  |
| 2 | 27705422 | 1 | [rs3749147](http://www.broadinstitute.org/mammals/haploreg/detail.php?query=&id=rs3749147) | G | A | 53 cell types | 39 bound proteins | Klf7,YY1,PU.1 | ZNF512 |  |
| 2 | 165221337 | 1 | [rs10195252](http://www.broadinstitute.org/mammals/haploreg/detail.php?query=&id=rs10195252) | T | C |  |  |  | COBLL1 |  |
| 2 | 27584444 | 1 | [rs1260326](http://www.broadinstitute.org/mammals/haploreg/detail.php?query=&id=rs1260326) | T | C |  |  | NRSF | GCKR |  |
| 2 | 27596107 | 1 | [rs780093](http://www.broadinstitute.org/mammals/haploreg/detail.php?query=&id=rs780093) | T | C | MCF-7 |  |  | GCKR |  |
| 2 | 27594741 | 1 | [rs780094](http://www.broadinstitute.org/mammals/haploreg/detail.php?query=&id=rs780094) | T | C | HepG2 | FOXA2,RXRA,MAFK | MafB | GCKR | Mafb |
| 3 | 12368125 | 1 | [rs1801282](http://www.broadinstitute.org/mammals/haploreg/detail.php?query=&id=rs1801282) | C | G |  |  |  | PPARG |  |
| 3 | 12368125 | 1 | [rs1801282](http://www.broadinstitute.org/mammals/haploreg/detail.php?query=&id=rs1801282) | C | G |  |  |  | PPARG |  |
| 3 | 188133484 | 1 | [rs10937275](http://www.broadinstitute.org/mammals/haploreg/detail.php?query=&id=rs10937275) | A | G | 4 cell types | 6 bound proteins |  | ST6GAL1 |  |
| 4 | 123417885 | 1 | [rs6534347](http://www.broadinstitute.org/mammals/haploreg/detail.php?query=&id=rs6534347) | A | G |  |  | AIRE | KIAA1109 |  |
| 4 | 7270834 | 1 | [rs4234798](http://www.broadinstitute.org/mammals/haploreg/detail.php?query=&id=rs4234798) | T | G | 41 cell types | CEBPB |  | SORCS2 |  |
| 4 | 118312585 | 1 | [rs10433903](http://www.broadinstitute.org/mammals/haploreg/detail.php?query=&id=rs10433903) | T | C |  | MAFK |  | 86kb 5' of TRAM1L1 |  |
| 5 | 132656783 | 1 | [rs17166496](http://www.broadinstitute.org/mammals/haploreg/detail.php?query=&id=rs17166496) | G | C | iPS | YY1 |  | FSTL4 |  |
| 6 | 11087374 | 1 | [rs12708716](http://www.broadinstitute.org/mammals/haploreg/detail.php?query=&id=rs12708716) | A | G | 5 cell types |  |  | CLEC16A |  |
| 6 | 39392028 | 1 | [rs1535500](http://www.broadinstitute.org/mammals/haploreg/detail.php?query=&id=rs1535500) | G | T | 14 cell types |  | MZF1::1-4 | KCNK16 |  |
| 6 | 126740412 | 1 | [rs9388489](http://www.broadinstitute.org/mammals/haploreg/detail.php?query=&id=rs9388489) | A | G |  |  | DMRT7 | 81bp 3' of AL356534.1 | DMRT7 |
| 6 | 32778233 | 1 | [rs113004881](http://www.broadinstitute.org/mammals/haploreg/detail.php?query=&id=rs113004881) | C | T |  |  | Crx | 16kb 5' of XXbac-BPG254F23.7 | CRX, rs2856718 |
| 6 | 31474574 | 1 | [rs115117173](http://www.broadinstitute.org/mammals/haploreg/detail.php?query=&id=rs115117173) | C | T |  |  | Hoxd10,Hoxa10,Hoxb13 | 965bp 5' of MICA |  |
| 6 | 24549725 | 1 | [rs9467160](http://www.broadinstitute.org/mammals/haploreg/detail.php?query=&id=rs9467160) | G | A | 9 cell types | CEBPB |  | GPLD1 |  |
| 6 | 32740810 | 1 | [rs9274407](http://www.broadinstitute.org/mammals/haploreg/detail.php?query=&id=rs9274407) | A | T | 15 cell types | POL2 |  | HLA-DQB1 |  |
| 7 | 28162747 | 1 | [rs849134](http://www.broadinstitute.org/mammals/haploreg/detail.php?query=&id=rs849134) | A | G |  |  | Pou2f1 | JAZF1 | OCT1 |
| 7 | 46720078 | 1 | [rs700752](http://www.broadinstitute.org/mammals/haploreg/detail.php?query=&id=rs700752) | C | G | 12 cell types |  |  | AC011294.3 |  |
| 7 | 44545705 | 1 | [rs2072183](http://www.broadinstitute.org/mammals/haploreg/detail.php?query=&id=rs2072183) | G | C | 6 cell types |  |  | NPC1L1 |  |
| 7 | 72648378 | 1 | [rs13247874](http://www.broadinstitute.org/mammals/haploreg/detail.php?query=&id=rs13247874) | C | T | 8 cell types |  | Evi-1 | MLXIPL | RUNX1 |
| 7 | 72664314 | 1 | [rs17145750](http://www.broadinstitute.org/mammals/haploreg/detail.php?query=&id=rs17145750) | C | T |  |  | PPAR | MLXIPL | PPARa:RXRa |
| 7 | 72620810 | 1 | [rs17145738](http://www.broadinstitute.org/mammals/haploreg/detail.php?query=&id=rs17145738) | C | T | 5 cell types |  | Msx-1 | 1.1kb 3' of TBL2 | MSX-1 |
| 8 | 96029687 | 1 | [rs896854](http://www.broadinstitute.org/mammals/haploreg/detail.php?query=&id=rs896854) | T | C | 9 cell types |  |  | C8orf38 |  |
| 8 | 9221006 | 1 | [rs4841132](http://www.broadinstitute.org/mammals/haploreg/detail.php?query=&id=rs4841132) | A | G |  |  |  | RP11-115J16.1 |  |
| 8 | 9222556 | 1 | [rs2126259](http://www.broadinstitute.org/mammals/haploreg/detail.php?query=&id=rs2126259) | T | C |  |  |  | RP11-115J16.1 |  |
| 8 | 9220768 | 1 | [rs9987289](http://www.broadinstitute.org/mammals/haploreg/detail.php?query=&id=rs9987289) | A | G |  |  |  | RP11-115J16.1 |  |
| 8 | 126551259 | 1 | [rs2954021](http://www.broadinstitute.org/mammals/haploreg/detail.php?query=&id=rs2954021) | A | G |  |  | XBP-1 | RP11-136O12.2 | ZSCAN4 |
| 8 | 145115531 | 1 | [rs11136341](http://www.broadinstitute.org/mammals/haploreg/detail.php?query=&id=rs11136341) | A | G | 23 cell types |  |  | PLEC |  |
| 8 | 126551259 | 1 | [rs2954021](http://www.broadinstitute.org/mammals/haploreg/detail.php?query=&id=rs2954021) | A | G |  |  | XBP-1 | RP11-136O12.2 | Zscan4 |
| 8 | 54235034 | 1 | [rs4503880](http://www.broadinstitute.org/mammals/haploreg/detail.php?query=&id=rs4503880) | T | C | 49 cell types |  |  | 15kb 3' of NEDD4L |  |
| 8 | 9215678 | 1 | [rs6984305](http://www.broadinstitute.org/mammals/haploreg/detail.php?query=&id=rs6984305) | A | T | 45 cell types | 4 bound proteins | CTCF | RP11-115J16.1 |  |
| 8 | 9220768 | 1 | [rs9987289](http://www.broadinstitute.org/mammals/haploreg/detail.php?query=&id=rs9987289) | A | G |  |  |  | RP11-115J16.1 |  |
| 8 | 126547160 | 1 | [rs2001945](http://www.broadinstitute.org/mammals/haploreg/detail.php?query=&id=rs2001945) | G | C | 4 cell types |  |  | 1.2kb 5' of RP11-136O12.2 |  |
| 9 | 4277466 | 1 | [rs7041847](http://www.broadinstitute.org/mammals/haploreg/detail.php?query=&id=rs7041847) | A | G | HRGEC,LNCaP |  |  | GLIS3 |  |
| 9 | 22019547 | 1 | [rs564398](http://www.broadinstitute.org/mammals/haploreg/detail.php?query=&id=rs564398) | T | C |  |  |  | MTAP | OST2 |
| 9 | 135143989 | 1 | [rs579459](http://www.broadinstitute.org/mammals/haploreg/detail.php?query=&id=rs579459) | T | C | 4 cell types | NFYA,POL2 |  | 3.6kb 5' of ABO |  |
| 9 | 103263054 | 1 | [rs10819937](http://www.broadinstitute.org/mammals/haploreg/detail.php?query=&id=rs10819937) | C | G | HNPCEpiC |  | NRSF | 7.5kb 5' of RP11-490D19.6 | NRSF |
| 10 | 6430456 | 1 | [rs947474](http://www.broadinstitute.org/mammals/haploreg/detail.php?query=&id=rs947474) | G | A | 10 cell types | NFKB |  | 1.8kb 5' of AL137145.1 |  |
| 10 | 114744078 | 1 | [rs7901695](http://www.broadinstitute.org/mammals/haploreg/detail.php?query=&id=rs7901695) | T | C |  |  | Hoxd10 | AL158212.1 | HOXD10 |
| 10 | 6134703 | 1 | [rs61839660](http://www.broadinstitute.org/mammals/haploreg/detail.php?query=&id=rs61839660) | C | T | 15 cell types | 11 bound proteins | FOXI1, FOXJ2, NKXA, FOXA, DOBOX4 | IL2RA | DOBOX4 |
| 10 | 73519645 | 1 | [rs1245541](http://www.broadinstitute.org/mammals/haploreg/detail.php?query=&id=rs1245541) | G | A | 9 cell types | CEBPB,USF2 |  | 848bp 5' of SPOCK2 |  |
| 10 | 64803828 | 1 | [rs7923609](http://www.broadinstitute.org/mammals/haploreg/detail.php?query=&id=rs7923609) | A | G |  |  |  | JMJD1C |  |
| 10 | 71258510 | 1 | [rs1227756](http://www.broadinstitute.org/mammals/haploreg/detail.php?query=&id=rs1227756) | G | A | GM12891 |  | TEF | COL13A1 | TEF |
| 10 | 72002446 | 1 | [rs10999409](http://www.broadinstitute.org/mammals/haploreg/detail.php?query=&id=rs10999409) | T | C | HCT-116,HRPEpiC |  |  | 4.2kb 3' of KIAA1274 |  |
| 10 | 64697616 | 1 | [rs10761731](http://www.broadinstitute.org/mammals/haploreg/detail.php?query=&id=rs10761731) | A | T | 47 cell types | 9 bound proteins |  | JMJD1C |  |
| 10 | 94829632 | 1 | [rs2068888](http://www.broadinstitute.org/mammals/haploreg/detail.php?query=&id=rs2068888) | G | A | 8 cell types | CEBPB | GATA | 2kb 3' of CYP26A1 | GATA1 |
| 11 | 17366148 | 1 | [rs5219](http://www.broadinstitute.org/mammals/haploreg/detail.php?query=&id=rs5219) | T | C | 4 cell types |  |  | KCNJ11 |  |
| 11 | 17365206 | 1 | [rs5215](http://www.broadinstitute.org/mammals/haploreg/detail.php?query=&id=rs5215) | C | T | 4 cell types | CTCF |  | KCNJ11 |  |
| 11 | 2138800 | 1 | [rs689](http://www.broadinstitute.org/mammals/haploreg/detail.php?query=&id=rs689) | A | T | 15 cell types |  | Ik-1 | INS | LYF-1 |
| 11 | 92331180 | 1 | [rs2166706](http://www.broadinstitute.org/mammals/haploreg/detail.php?query=&id=rs2166706) | T | C |  |  |  | 7.4kb 5' of RP11-676F20.1 |  |
| 11 | 116157633 | 1 | [rs6589566](http://www.broadinstitute.org/mammals/haploreg/detail.php?query=&id=rs6589566) | G | A |  |  | XBP-1 | ZNF259 | XBP1, HTF |
| 11 | 116154127 | 1 | [rs964184](http://www.broadinstitute.org/mammals/haploreg/detail.php?query=&id=rs964184) | G | C | HAEpiC,HVMF |  | Foxj2 | ZNF259 | FOXJ2 |
| 11 | 116112647 | 1 | [rs1558861](http://www.broadinstitute.org/mammals/haploreg/detail.php?query=&id=rs1558861) | C | T | 8988T |  |  | 11kb 3' of BUD13 |  |
| 11 | 116167789 | 1 | [rs651821](http://www.broadinstitute.org/mammals/haploreg/detail.php?query=&id=rs651821) | C | T | 5 cell types |  |  | APOA5 |  |
| 11 | 65106332 | 1 | [rs6591182](http://www.broadinstitute.org/mammals/haploreg/detail.php?query=&id=rs6591182) | T | G | 12 cell types |  | OLF1, PPAR,SZF1, MYF | EHBP1L1 | PPARa:RXRa, Myf6 |
| 11 | 116167789 | 1 | [rs651821](http://www.broadinstitute.org/mammals/haploreg/detail.php?query=&id=rs651821) | C | T | 5 cell types |  |  | APOA5 |  |
| 11 | 116154127 | 1 | [rs964184](http://www.broadinstitute.org/mammals/haploreg/detail.php?query=&id=rs964184) | G | C | HAEpiC,HVMF |  | Foxj2 | ZNF259 | FOXJ2 |
| 11 | 116165896 | 1 | [rs2266788](http://www.broadinstitute.org/mammals/haploreg/detail.php?query=&id=rs2266788) | G | A | 8 cell types |  |  | APOA5 |  |
| 11 | 116237722 | 1 | [rs2075292](http://www.broadinstitute.org/mammals/haploreg/detail.php?query=&id=rs2075292) | G | T | 58 cell types | 5 bound proteins |  | SIK3 |  |
| 11 | 116112647 | 1 | [rs1558861](http://www.broadinstitute.org/mammals/haploreg/detail.php?query=&id=rs1558861) | C | T | 8988T |  |  | 11kb 3' of BUD13 |  |
| 11 | 116158506 | 1 | [rs2075290](http://www.broadinstitute.org/mammals/haploreg/detail.php?query=&id=rs2075290) | C | T |  |  | Nkx6-2 | ZNF259 |  |
| 12 | 110971201 | 1 | [rs17696736](http://www.broadinstitute.org/mammals/haploreg/detail.php?query=&id=rs17696736) | A | G |  |  | Mrg,RREB-1,Tgif1 | NAA25 | MRG1 |
| 12 | 54768447 | 1 | [rs2292239](http://www.broadinstitute.org/mammals/haploreg/detail.php?query=&id=rs2292239) | T | G | GM12864,WERI-Rb-1 | CTCF,ZNF263 |  | ERBB3 |  |
| 12 | 110368991 | 1 | [rs3184504](http://www.broadinstitute.org/mammals/haploreg/detail.php?query=&id=rs3184504) | T | C | NHEK,WI-38 |  | HES1,Mtf1 | SH2B3 | MTF1 |
| 12 | 101399699 | 1 | [rs35767](http://www.broadinstitute.org/mammals/haploreg/detail.php?query=&id=rs35767) | A | G | MCF-7,T-47D,HCFaa | EBF1 |  | 1.1kb 5' of IGF1 | SRF, TCF3,Tcfap2e |
| 12 | 119901033 | 1 | [rs1169288](http://www.broadinstitute.org/mammals/haploreg/detail.php?query=&id=rs1169288) | A | C | 7 cell types | POL2, TAF1 | NRSF | C21orf81 |  |
| 12 | 3627809 | 1 | [rs887304](http://www.broadinstitute.org/mammals/haploreg/detail.php?query=&id=rs887304) | T | C |  |  | GLI | EFCAB4B | GLI, GLI1 |
| 13 | 28176450 | 1 | [rs1305088](http://www.broadinstitute.org/mammals/haploreg/detail.php?query=&id=rs1305088) | T | C |  |  |  | SLC46A3 |  |
| 13 | 52962982 | 1 | [rs9568856](http://www.broadinstitute.org/mammals/haploreg/detail.php?query=&id=rs9568856) | G | A |  |  | Six | 123kb 5' of AL450423.1 |  |
| 14 | 68333352 | 1 | [rs1465788](http://www.broadinstitute.org/mammals/haploreg/detail.php?query=&id=rs1465788) | T | C | 5 cell types |  |  | 408bp 5' of C14orf181 |  |
| 14 | 89370788 | 1 | [rs12100561](http://www.broadinstitute.org/mammals/haploreg/detail.php?query=&id=rs12100561) | A | G |  |  |  | EFCAB11 |  |
| 14 | 23953727 | 1 | [rs8017377](http://www.broadinstitute.org/mammals/haploreg/detail.php?query=&id=rs8017377) | G | A | GM12891 |  |  |  |  |
| 15 | 36625556 | 1 | [rs8035957](http://www.broadinstitute.org/mammals/haploreg/detail.php?query=&id=rs8035957) | T | C |  |  | Gfi1 | RASGRP1 |  |
| 15 | 75534245 | 1 | [rs7178572](http://www.broadinstitute.org/mammals/haploreg/detail.php?query=&id=rs7178572) | A | G |  |  |  | HMG20A |  |
| 15 | 78219277 | 1 | [rs11634397](http://www.broadinstitute.org/mammals/haploreg/detail.php?query=&id=rs11634397) | A | G |  |  | CHX10,Ncx,Dlx3 | 1.5kb 3' of ZFAND6 | NKX1-2 |
| 15 | 68896201 | 1 | [rs1549318](http://www.broadinstitute.org/mammals/haploreg/detail.php?query=&id=rs1549318) | C | T |  |  |  | 15kb 3' of LARP6 |  |
| 15 | 58670573 | 1 | [rs339969](http://www.broadinstitute.org/mammals/haploreg/detail.php?query=&id=rs339969) | C | A | 49 cell types | 14 bound proteins | RREB-1 | RORA |  |
| 15 | 56470658 | 1 | [rs1532085](http://www.broadinstitute.org/mammals/haploreg/detail.php?query=&id=rs1532085) | A | G |  |  |  | 19kb 5' of LIPC |  |
| 16 | 1778837 | 1 | [rs1065656](http://www.broadinstitute.org/mammals/haploreg/detail.php?query=&id=rs1065656) | C | G | 19 cell types |  |  | NUBP2 |  |
| 16 | 15056147 | 1 | [rs11075253](http://www.broadinstitute.org/mammals/haploreg/detail.php?query=&id=rs11075253) | C | A | 5 cell types |  | GATA,En-1 | NTAN1 | GATA1, GATA-2,GATA5,GATA6, Tal1::gata1 |
| 16 | 77509940 | 1 | [rs9923451](http://www.broadinstitute.org/mammals/haploreg/detail.php?query=&id=rs9923451) | A | G |  |  |  | WWOX |  |
| 16 | 52361075 | 1 | [rs1558902](http://www.broadinstitute.org/mammals/haploreg/detail.php?query=&id=rs1558902) | T | A |  |  |  | FTO |  |
| 16 | 52366748 | 1 | [rs1121980](http://www.broadinstitute.org/mammals/haploreg/detail.php?query=&id=rs1121980) | G | A | NB4 | PU1,USF1 |  | FTO |  |
| 16 | 52358455 | 1 | [rs1421085](http://www.broadinstitute.org/mammals/haploreg/detail.php?query=&id=rs1421085) | T | C |  |  | HNF6 | FTO | HNF6 |
| 16 | 55552737 | 1 | [rs1800775](http://www.broadinstitute.org/mammals/haploreg/detail.php?query=&id=rs1800775) | C | A | 10 cell types | AP2GAMMA |  | 525bp 5' of CETP | ZFP740 |
| 17 | 35319766 | 1 | [rs2290400](http://www.broadinstitute.org/mammals/haploreg/detail.php?query=&id=rs2290400) | T | C |  |  | BLIMP1 | GSDMB | BLIMP1 |
| 17 | 44024429 | 1 | [rs9299](http://www.broadinstitute.org/mammals/haploreg/detail.php?query=&id=rs9299) | C | T | 5 cell types | SUZ12 | Tcf | HOXB5 |  |
| 19 | 44430627 | 1 | [rs12979860](http://www.broadinstitute.org/mammals/haploreg/detail.php?query=&id=rs12979860) | C | T | 33 cell types |  |  | 3.1kb 5' of IL28B | DEAF1 |
| 19 | 38601550 | 1 | [rs8182584](http://www.broadinstitute.org/mammals/haploreg/detail.php?query=&id=rs8182584) | T | G | 7 cell types |  |  | PEPD |  |
| 19 | 11056030 | 1 | [rs11668477](http://www.broadinstitute.org/mammals/haploreg/detail.php?query=&id=rs11668477) | A | G | 6 cell types |  | EWSR1-FLI1 | 5kb 5' of LDLR |  |
| 19 | 50103919 | 1 | [rs7412](http://www.broadinstitute.org/mammals/haploreg/detail.php?query=&id=rs7412) | C | T | Chorion,H1-hESC,iPS |  |  | APOE |  |
| 19 | 50106291 | 1 | [rs439401](http://www.broadinstitute.org/mammals/haploreg/detail.php?query=&id=rs439401) | T | C | 46 cell types | 7 bound proteins | UF1H3BETA,Sp1,VDR | 1.8kb 3' of APOE | VDR |
| 20 | 43968058 | 1 | [rs6065904](http://www.broadinstitute.org/mammals/haploreg/detail.php?query=&id=rs6065904) | G | A | 11 cell types | AP2GAMMA,BAF155,GTF2F1 | HNF4 | PLTP |  |
| 20 | 14275899 | 1 | [rs6079395](http://www.broadinstitute.org/mammals/haploreg/detail.php?query=&id=rs6079395) | G | A |  |  | EWSR1-FLI1,Foxc1 | MACROD2 |  |
| 20 | 44009909 | 1 | [rs7679](http://www.broadinstitute.org/mammals/haploreg/detail.php?query=&id=rs7679) | T | C | GM12892,LNCaP |  | GATA | PCIF1 | GATA-1 |
| 21 | 30068040 | 1 | [rs455804](http://www.broadinstitute.org/mammals/haploreg/detail.php?query=&id=rs455804) | A | C |  |  | PPARg | GRIK1 | PPARg:RXRa |
| 22 | 22625286 | 1 | [rs2739330](http://www.broadinstitute.org/mammals/haploreg/detail.php?query=&id=rs2739330) | T | C |  |  |  | 4.3kb 3' of GSTT2B |  |

Table S4: Novel SNPs identified in higher control peaks than ASH.

| **Chromosome** | **Position** | **Reference** | **Change** | **Quality** | **Coverage** | **Gene_ID** | **Gene name** | **Trancript_ID** | **Effect** |
| --- | --- | --- | --- | --- | --- | --- | --- | --- | --- |
| 1 | 16906960 | G | A | 129 | 69 | ENSG00000116219 | ESPNP | ENST00000270691 | INTRON |
| 16 | 57054799 | G | T | 72 | 18 | ENSG00000103034 | NDRG4 | ENST00000394282 | UPSTREAM: 924 bases |
| 16 | 57054799 | G | T | 72 | 18 | ENSG00000103034 | NDRG4 | ENST00000258187 | UPSTREAM: 319 bases |
| 1 | 205164090 | G | A | 137 | 37 | ENSG00000162894 | FAIM3 | ENST00000367091 | UPSTREAM: 2124 bases |
| 1 | 205164090 | G | A | 137 | 37 | ENSG00000162896 | PIGR | ENST00000356495 | DOWNSTREAM: 4405 bases |
| 1 | 205164060 | A | T | 177 | 32 | ENSG00000162894 | FAIM3 | ENST00000367091 | UPSTREAM: 2094 bases |
| 1 | 205164060 | A | T | 177 | 32 | ENSG00000162896 | PIGR | ENST00000356495 | DOWNSTREAM: 4435 bases |
| 9 | 67908931 | C | A | 63 | 23 | ENSG00000217091 | RP11-764K9.2 | ENST00000407998 | UPSTREAM: 4915 bases |
| 9 | 67908876 | T | C | 128 | 21 | ENSG00000217091 | RP11-764K9.2 | ENST00000407998 | UPSTREAM: 4860 bases |
| 17 | 46367626 | G | C | 88 | 19 |  |  |  | INTERGENIC |
| 7 | 149331527 | C | T | 93 | 13 |  |  |  | INTERGENIC |
| 17 | 28173539 | A | G | 175 | 64 | ENSG00000176658 | MYO1D | ENST00000318217 | INTRON |
| 17 | 28173539 | A | G | 175 | 64 | ENSG00000176658 | MYO1D | ENST00000394649 | INTRON |
| 8 | 11669165 | C | T | 124 | 11 | ENSG00000154328 | NEIL2 | ENST00000382309 | INTRON |
| 8 | 11669165 | C | T | 124 | 11 | ENSG00000154328 | NEIL2 | ENST00000403422 | INTRON |
| 8 | 11669165 | C | T | 124 | 11 | ENSG00000154328 | NEIL2 | ENST00000354579 | INTRON |
| 8 | 11669165 | C | T | 124 | 11 | ENSG00000154328 | NEIL2 | ENST00000284503 | INTRON |
| 19 | 2526622 | T | C | 98 | 19 | ENSG00000176533 | AC005512.1-2 | ENST00000382159 | INTRON |
| 6 | 117909941 | A | G | 157 | 19 | ENSG00000164465 | DCBLD1 | ENST00000368503 | UPSTREAM: 572 bases |
| 6 | 117909941 | A | G | 157 | 19 | ENSG00000164465 | DCBLD1 | ENST00000392504 | UPSTREAM: 572 bases |
| 6 | 117909941 | A | G | 157 | 19 | ENSG00000164465 | DCBLD1 | ENST00000338728 | UPSTREAM: 572 bases |
| 6 | 117909941 | A | G | 157 | 19 | ENSG00000164465 | DCBLD1 | ENST00000296955 | UPSTREAM: 572 bases |
| 19 | 45664338 | C | T | 60 | 56 | ENSG00000090013 | BLVRB | ENST00000263368 | UPSTREAM: 821 bases |
| 19 | 45664338 | C | T | 60 | 56 | ENSG00000160460 | SPTBN4 | ENST00000344104 | UPSTREAM: 628 bases |
| 19 | 45664338 | C | T | 60 | 56 | ENSG00000160460 | SPTBN4 | ENST00000338932 | UPSTREAM: 628 bases |
| 19 | 45664338 | C | T | 60 | 56 | ENSG00000160460 | SPTBN4 | ENST00000352632 | UPSTREAM: 628 bases |
| 1 | 200253130 | G | A | 98 | 27 | ENSG00000163435 | ELF3 | ENST00000367283 | DOWNSTREAM: 1370 bases |
| 1 | 200253130 | G | A | 98 | 27 | ENSG00000163435 | ELF3 | ENST00000310044 | DOWNSTREAM: 1376 bases |
| 1 | 200253130 | G | A | 98 | 27 | ENSG00000163435 | ELF3 | ENST00000367284 | DOWNSTREAM: 1376 bases |
| 1 | 200253130 | G | A | 98 | 27 | ENSG00000163435 | ELF3 | ENST00000359651 | DOWNSTREAM: 1376 bases |
| 20 | 42467066 | G | A | 105 | 31 | ENSG00000101076 | HNF4A | ENST00000372920 | INTRON |
| 20 | 42467066 | G | A | 105 | 31 | ENSG00000101076 | HNF4A | ENST00000316673 | INTRON |
| 20 | 42467066 | G | A | 105 | 31 | ENSG00000101076 | HNF4A | ENST00000338692 | INTRON |
| 20 | 42467066 | G | A | 105 | 31 | ENSG00000101076 | HNF4A | ENST00000372913 | INTRON |
| 20 | 42467066 | G | A | 105 | 31 | ENSG00000101076 | HNF4A | ENST00000316099 | INTRON |
| 1 | 142123527 | T | C | 70 | 61 |  |  |  | INTERGENIC |
| 4 | 1787535 | A | C | 51 | 20 | ENSG00000168924 | LETM1 | ENST00000302787 | INTRON |
| 1 | 142123596 | A | C | 52 | 57 |  |  |  | INTERGENIC |
| 9 | 67909177 | T | C | 123 | 35 |  |  |  | INTERGENIC |
| 13 | 110065331 | A | G | 180 | 27 | ENSG00000213995 | CARKD | ENST00000309957 | UPSTREAM: 678 bases |
| 13 | 110065331 | A | G | 180 | 27 | ENSG00000213995 | CARKD | ENST00000397191 | UPSTREAM: 551 bases |
| 7 | 149331579 | A | C | 127 | 18 |  |  |  | INTERGENIC |
| 9 | 67909064 | C | T | 89 | 33 |  |  |  | INTERGENIC |
| 9 | 67908829 | G | C | 136 | 26 | ENSG00000217091 | RP11-764K9.2 | ENST00000407998 | UPSTREAM: 4813 bases |
| 13 | 20770836 | C | T | 225 | 75 | ENSG00000218629 | RP11-101P17.8 | ENST00000403153 | TRANSCRIPT: ENST00000403153 |
| 17 | 76222913 | T | C | 78 | 10 | ENSG00000141564 | AC127496.5-2 | ENST00000306801 | INTRON |
| 8 | 92121756 | T | C | 151 | 49 | ENSG00000155099 | TMEM55A | ENST00000285419 | INTRON |
| 17 | 17339852 | G | A | 184 | 95 | ENSG00000141026 | MED9 | ENST00000268711 | DOWNSTREAM: 2593 bases |
| 17 | 17339852 | G | A | 184 | 95 | ENSG00000108551 | RASD1 | ENST00000225688 | INTRON |
| 9 | 116130467 | G | A | 113 | 30 | ENSG00000204154 | ORM2 | ENST00000374100 | UPSTREAM: 1539 bases |
| 9 | 116130467 | G | A | 113 | 30 | ENSG00000187681 | ORM1 | ENST00000259396 | DOWNSTREAM: 2009 bases |
| 22 | 23765550 | C | T | 80 | 12 | ENSG00000197077 | CTA-221G9.5 | ENST00000406486 | NON_SYNONYMOUS_CODING |
| 22 | 23765550 | C | T | 80 | 12 | ENSG00000197077 | CTA-221G9.5 | ENST00000358431 | NON_SYNONYMOUS_CODING |
| 9 | 67908976 | T | C | 96 | 15 | ENSG00000217091 | RP11-764K9.2 | ENST00000407998 | UPSTREAM: 4960 bases |
| 9 | 67908940 | T | C | 125 | 20 | ENSG00000217091 | RP11-764K9.2 | ENST00000407998 | UPSTREAM: 4924 bases |
| 13 | 114011417 | T | C | 90 | 15 |  |  |  | INTERGENIC |
| 1 | 1274353 | G | A | 105 | 39 | ENSG00000107404 | DVL1 | ENST00000263743 | INTRON |
| 1 | 1274353 | G | A | 105 | 39 | ENSG00000107404 | DVL1 | ENST00000345100 | UTR_5_PRIME: 45 bases from TSS |
| 1 | 1274353 | G | A | 105 | 39 | ENSG00000107404 | DVL1 | ENST00000378888 | UPSTREAM: 45 bases |
| 1 | 1274353 | G | A | 105 | 39 | ENSG00000107404 | DVL1 | ENST00000378891 | UPSTREAM: 45 bases |
| 1 | 1274353 | G | A | 105 | 39 | ENSG00000162576 | MXRA8 | ENST00000309212 | DOWNSTREAM: 3582 bases |
| 1 | 1274353 | G | A | 105 | 39 | ENSG00000162576 | MXRA8 | ENST00000342753 | DOWNSTREAM: 3582 bases |
| 1 | 1274353 | G | A | 105 | 39 | ENSG00000162576 | MXRA8 | ENST00000378864 | DOWNSTREAM: 3582 bases |
| 8 | 64084520 | C | T | 106 | 41 |  |  |  | INTERGENIC |
| 1 | 16906960 | G | A | 129 | 69 | ENSG00000116219 | ESPNP | ENST00000270691 | INTRON |
| 8 | 1909567 | C | G | 53 | 25 | ENSG00000176595 | KBTBD11 | ENST00000320248 | INTRON |
| 17 | 38756931 | G | A | 52 | 15 |  |  |  | INTERGENIC |
| 2 | 236243082 | G | C | 139 | 40 | ENSG00000157985 | AGAP1 | ENST00000402604 | INTRON |
| 2 | 236243082 | G | C | 139 | 40 | ENSG00000157985 | AGAP1 | ENST00000409538 | START_GAINED: CTG, UTR_5_PRIME: 401 bases from TSS |
| 2 | 236243082 | G | C | 139 | 40 | ENSG00000157985 | AGAP1 | ENST00000409457 | INTRON |
| 2 | 236243082 | G | C | 139 | 40 | ENSG00000157985 | AGAP1 | ENST00000304032 | INTRON |
| 2 | 236243082 | G | C | 139 | 40 | ENSG00000157985 | AGAP1 | ENST00000336665 | INTRON |
| 17 | 21097204 | G | A | 76 | 27 | ENSG00000154035 | C17orf103 | ENST00000399011 | UTR_5_PRIME: 35 bases from TSS |
| 17 | 21097204 | G | A | 76 | 27 | ENSG00000154035 | C17orf103 | ENST00000284177 | UPSTREAM: 35 bases |
| 20 | 26137704 | A | C | 64 | 13 | ENSG00000207985 | hsa-mir-663 | ENST00000385250 | UPSTREAM: 790 bases |
| 1 | 205164090 | G | A | 137 | 37 | ENSG00000162894 | FAIM3 | ENST00000367091 | UPSTREAM: 2124 bases |
| 1 | 205164090 | G | A | 137 | 37 | ENSG00000162896 | PIGR | ENST00000356495 | DOWNSTREAM: 4405 bases |
| 17 | 38756914 | A | G | 72 | 15 |  |  |  | INTERGENIC |
| 20 | 26137691 | A | C | 69 | 16 | ENSG00000207985 | hsa-mir-663 | ENST00000385250 | UPSTREAM: 777 bases |
| 17 | 38756874 | A | G | 94 | 58 |  |  |  | INTERGENIC |
| 1 | 205164060 | A | T | 177 | 32 | ENSG00000162894 | FAIM3 | ENST00000367091 | UPSTREAM: 2094 bases |
| 1 | 205164060 | A | T | 177 | 32 | ENSG00000162896 | PIGR | ENST00000356495 | DOWNSTREAM: 4435 bases |
| 19 | 62973058 | G | T | 82 | 52 | ENSG00000083828 | ZNF586 | ENST00000396154 | NON_SYNONYMOUS_CODING |
| 19 | 62973058 | G | T | 82 | 52 | ENSG00000083828 | ZNF586 | ENST00000396150 | NON_SYNONYMOUS_CODING |
| 19 | 62973058 | G | T | 82 | 52 | ENSG00000083828 | ZNF586 | ENST00000308137 | NON_SYNONYMOUS_CODING |
| 14 | 23528260 | C | T | 222 | 40 | ENSG00000187630 | DHRS4L2 | ENST00000335125 | INTRON |
| 14 | 23528260 | C | T | 222 | 40 | ENSG00000187630 | DHRS4L2 | ENST00000382755 | INTRON |
| 14 | 23528260 | C | T | 222 | 40 | ENSG00000187630 | DHRS4L2 | ENST00000397071 | INTRON |
| X | 2856702 | A | G | 124 | 19 | ENSG00000006756 | ARSD | ENST00000381154 | INTRON |
| X | 2856702 | A | G | 124 | 19 | ENSG00000006756 | ARSD | ENST00000217890 | INTRON |
| X | 2856702 | A | G | 124 | 19 | ENSG00000006756 | ARSD | ENST00000358177 | INTRON |
| 10 | 23767962 | C | A | 97 | 19 | ENSG00000165312 | OTUD1 | ENST00000376495 | UPSTREAM: 242 bases |
| 9 | 66194512 | A | G | 56 | 14 | ENSG00000218456 | RP11-262H14.10 | ENST00000405657 | UPSTREAM: 249 bases |
| 9 | 66194512 | A | G | 56 | 14 | ENSG00000202474 | 5S_rRNA | ENST00000365604 | DOWNSTREAM: 3451 bases |
| 11 | 128654890 | T | A | 197 | 95 |  |  |  | INTERGENIC |
| 2 | 231610560 | T | C | 68 | 30 | ENSG00000204128 | C2orf72 | ENST00000373640 | UPSTREAM: 346 bases |
| 19 | 15905682 | A | G | 64 | 56 | ENSG00000171903 | CYP4F11 | ENST00000402119 | INTRON |
| 19 | 15905682 | A | G | 64 | 56 | ENSG00000171903 | CYP4F11 | ENST00000326742 | INTRON |
| 19 | 15905682 | A | G | 64 | 56 | ENSG00000171903 | CYP4F11 | ENST00000248041 | INTRON |
| 2 | 25417194 | G | A | 70 | 63 | ENSG00000119772 | DNMT3A | ENST00000321117 | INTRON |
| 2 | 25417194 | G | A | 70 | 63 | ENSG00000119772 | DNMT3A | ENST00000406659 | INTRON |
| 2 | 25417194 | G | A | 70 | 63 | ENSG00000119772 | DNMT3A | ENST00000264709 | INTRON |
| 9 | 79836880 | C | A | 124 | 51 | ENSG00000156052 | GNAQ | ENST00000286548 | UPSTREAM: 868 bases |
| 11 | 809507 | G | A | 218 | 61 | ENSG00000177666 | PNPLA2 | ENST00000336615 | INTRON |
| 11 | 809507 | G | A | 218 | 61 | ENSG00000177666 | PNPLA2 | ENST00000397431 | UPSTREAM: 120 bases |
| 8 | 1909133 | G | A | 147 | 93 | ENSG00000176595 | KBTBD11 | ENST00000320248 | UPSTREAM: 318 bases |
| 7 | 149331527 | C | T | 93 | 13 |  |  |  | INTERGENIC |
| 17 | 38755160 | G | C | 68 | 48 |  |  |  | INTERGENIC |
| 17 | 28173539 | A | G | 175 | 64 | ENSG00000176658 | MYO1D | ENST00000318217 | INTRON |
| 17 | 28173539 | A | G | 175 | 64 | ENSG00000176658 | MYO1D | ENST00000394649 | INTRON |
| 1 | 47178257 | C | G | 225 | 29 | ENSG00000187048 | CYP4A11 | ENST00000310638 | INTRON |
| 1 | 47178257 | C | G | 225 | 29 | ENSG00000187048 | CYP4A11 | ENST00000371905 | INTRON |
| 1 | 47178257 | C | G | 225 | 29 | ENSG00000187048 | CYP4A11 | ENST00000371904 | INTRON |
| X | 71318218 | G | A | 222 | 14 | ENSG00000102309 | PIN4 | ENST00000218432 | UPSTREAM: 33 bases |
| X | 71318218 | G | A | 222 | 14 | ENSG00000102309 | PIN4 | ENST00000373669 | UPSTREAM: 33 bases |
| X | 71318218 | G | A | 222 | 14 | ENSG00000102309 | PIN4 | ENST00000373662 | UPSTREAM: 84 bases |
| 3 | 50316189 | C | A | 225 | 31 | ENSG00000214699 | NAT6 | ENST00000354862 | UPSTREAM: 4473 bases |
| 3 | 50316189 | C | A | 225 | 31 | ENSG00000186792 | HYAL3 | ENST00000359051 | UPSTREAM: 4286 bases |
| 3 | 50316189 | C | A | 225 | 31 | ENSG00000186792 | HYAL3 | ENST00000066014 | UPSTREAM: 4286 bases |
| 3 | 50316189 | C | A | 225 | 31 | ENSG00000186792 | HYAL3 | ENST00000336307 | UPSTREAM: 4286 bases |
| 3 | 50316189 | C | A | 225 | 31 | ENSG00000114378 | HYAL1 | ENST00000320295 | INTRON |
| 3 | 50316189 | C | A | 225 | 31 | ENSG00000114378 | HYAL1 | ENST00000395144 | UPSTREAM: 181 bases |
| 3 | 50316189 | C | A | 225 | 31 | ENSG00000114378 | HYAL1 | ENST00000395143 | UPSTREAM: 181 bases |
| 3 | 50316189 | C | A | 225 | 31 | ENSG00000114378 | HYAL1 | ENST00000266031 | UPSTREAM: 181 bases |
| 4 | 48863059 | G | A | 63 | 30 |  |  |  | INTERGENIC |
| 19 | 15905639 | A | G | 68 | 35 | ENSG00000171903 | CYP4F11 | ENST00000402119 | INTRON |
| 19 | 15905639 | A | G | 68 | 35 | ENSG00000171903 | CYP4F11 | ENST00000326742 | INTRON |
| 19 | 15905639 | A | G | 68 | 35 | ENSG00000171903 | CYP4F11 | ENST00000248041 | INTRON |
| 1 | 231816959 | C | A | 109 | 30 | ENSG00000135750 | KCNK1 | ENST00000366621 | INTRON |
| 19 | 3045215 | C | G | 50 | 16 | ENSG00000088256 | GNA11 | ENST00000078429 | UPSTREAM: 193 bases |
| 20 | 26137499 | A | G | 95 | 30 | ENSG00000207985 | hsa-mir-663 | ENST00000385250 | UPSTREAM: 585 bases |
| 17 | 38755097 | A | G | 62 | 25 |  |  |  | INTERGENIC |
| 1 | 142123527 | T | C | 70 | 61 |  |  |  | INTERGENIC |
| 14 | 73074261 | A | G | 222 | 52 | ENSG00000187105 | HEATR4 | ENST00000334988 | INTRON |
| 14 | 73074261 | A | G | 222 | 52 | ENSG00000184227 | ACOT1 | ENST00000311148 | NON_SYNONYMOUS_CODING |
| 17 | 38755091 | A | G | 79 | 23 |  |  |  | INTERGENIC |
| 19 | 15905695 | A | C | 88 | 57 | ENSG00000171903 | CYP4F11 | ENST00000402119 | INTRON |
| 19 | 15905695 | A | C | 88 | 57 | ENSG00000171903 | CYP4F11 | ENST00000326742 | INTRON |
| 19 | 15905695 | A | C | 88 | 57 | ENSG00000171903 | CYP4F11 | ENST00000248041 | INTRON |
| 19 | 15905695 | A | G | 88 | 57 | ENSG00000171903 | CYP4F11 | ENST00000402119 | INTRON |
| 19 | 15905695 | A | G | 88 | 57 | ENSG00000171903 | CYP4F11 | ENST00000326742 | INTRON |
| 19 | 15905695 | A | G | 88 | 57 | ENSG00000171903 | CYP4F11 | ENST00000248041 | INTRON |
| 1 | 142123596 | A | C | 52 | 57 |  |  |  | INTERGENIC |
| 6 | 167933542 | A | G | 94 | 13 | ENSG00000146521 | C6orf123 | ENST00000366822 | INTRON |
| 6 | 167933542 | A | G | 94 | 13 | ENSG00000146521 | C6orf123 | ENST00000340619 | INTRON |
| 6 | 31347130 | A | G | 222 | 78 | ENSG00000204525 | HLA-C | ENST00000376237 | INTRON |
| 6 | 31347130 | A | G | 222 | 78 | ENSG00000204525 | HLA-C | ENST00000376235 | INTRON |
| 6 | 31347130 | A | G | 222 | 78 | ENSG00000204525 | HLA-C | ENST00000396254 | INTRON |
| 6 | 31347130 | A | G | 222 | 78 | ENSG00000204525 | HLA-C | ENST00000406626 | INTRON |
| 6 | 31347130 | A | G | 222 | 78 | ENSG00000214892 | AL671883.3-1 | ENST00000399193 | UPSTREAM: 4208 bases |
| 6 | 31347130 | A | C | 222 | 78 | ENSG00000204525 | HLA-C | ENST00000376237 | INTRON |
| 6 | 31347130 | A | C | 222 | 78 | ENSG00000204525 | HLA-C | ENST00000376235 | INTRON |
| 6 | 31347130 | A | C | 222 | 78 | ENSG00000204525 | HLA-C | ENST00000396254 | INTRON |
| 6 | 31347130 | A | C | 222 | 78 | ENSG00000204525 | HLA-C | ENST00000406626 | INTRON |
| 6 | 31347130 | A | C | 222 | 78 | ENSG00000214892 | AL671883.3-1 | ENST00000399193 | UPSTREAM: 4208 bases |
| 17 | 38756821 | T | C | 82 | 86 |  |  |  | INTERGENIC |
| 17 | 38756966 | G | C | 66 | 15 |  |  |  | INTERGENIC |
| 19 | 15905611 | T | C | 55 | 24 | ENSG00000171903 | CYP4F11 | ENST00000402119 | INTRON |
| 19 | 15905611 | T | C | 55 | 24 | ENSG00000171903 | CYP4F11 | ENST00000326742 | INTRON |
| 19 | 15905611 | T | C | 55 | 24 | ENSG00000171903 | CYP4F11 | ENST00000248041 | INTRON |
| 21 | 43268428 | G | A | 156 | 76 | ENSG00000160199 | PKNOX1 | ENST00000291547 | INTRON |
| 20 | 26137698 | A | C | 71 | 13 | ENSG00000207985 | hsa-mir-663 | ENST00000385250 | UPSTREAM: 784 bases |
| 7 | 149331579 | A | C | 127 | 18 |  |  |  | INTERGENIC |
| 9 | 79836879 | G | A | 120 | 51 | ENSG00000156052 | GNAQ | ENST00000286548 | UPSTREAM: 867 bases |
| 17 | 21097525 | T | G | 134 | 60 | ENSG00000154035 | C17orf103 | ENST00000284177 | UPSTREAM: 356 bases |
| 17 | 21097525 | T | G | 134 | 60 | ENSG00000154035 | C17orf103 | ENST00000399011 | UPSTREAM: 210 bases |
| 6 | 31432574 | C | G | 107 | 84 | ENSG00000204525 | HLA-C | ENST00000376228 | SYNONYMOUS_CODING |
| 6 | 31432574 | C | G | 107 | 84 | ENSG00000204525 | HLA-C | ENST00000406626 | INTRON |
| 6 | 30002372 | C | G | 129 | 70 | ENSG00000219322 | HLA-K | ENST00000406831 | TRANSCRIPT: ENST00000406831 |
| 6 | 30002372 | C | G | 129 | 70 | ENSG00000181573 | HCG4P6 | ENST00000402127 | TRANSCRIPT: ENST00000402127 |
| 17 | 38755158 | A | C | 70 | 48 |  |  |  | INTERGENIC |
| 2 | 114077967 | C | T | 225 | 64 | ENSG00000155352 | FAM39B | ENST00000285718 | DOWNSTREAM: 4888 bases |
| 2 | 114077967 | C | T | 225 | 64 | ENSG00000213256 | AL078621.19-1 | ENST00000393169 | UPSTREAM: 4503 bases |
| 17 | 38756838 | T | C | 96 | 90 |  |  |  | INTERGENIC |
| 19 | 15905651 | T | C | 76 | 40 | ENSG00000171903 | CYP4F11 | ENST00000402119 | INTRON |
| 19 | 15905651 | T | C | 76 | 40 | ENSG00000171903 | CYP4F11 | ENST00000326742 | INTRON |
| 19 | 15905651 | T | C | 76 | 40 | ENSG00000171903 | CYP4F11 | ENST00000248041 | INTRON |
| 7 | 99411341 | C | A | 225 | 50 | ENSG00000160862 | AZGP1 | ENST00000292401 | INTRON |
| 7 | 99411341 | C | A | 225 | 50 | ENSG00000214313 | AC004522.3-1 | ENST00000398087 | UPSTREAM: 4984 bases |
| 17 | 38756927 | A | G | 50 | 16 |  |  |  | INTERGENIC |
| 6 | 158573205 | G | A | 73 | 20 |  |  |  | INTERGENIC |
| 8 | 1909121 | A | C | 149 | 83 | ENSG00000176595 | KBTBD11 | ENST00000320248 | UPSTREAM: 330 bases |
| 22 | 20745596 | T | G | 68 | 14 | ENSG00000217320 | IGLVIV-66-1 | ENST00000406817 | DOWNSTREAM: 2292 bases |
| 22 | 20745596 | T | G | 68 | 14 | ENSG00000220863 | IGLVV-66 | ENST00000402961 | TRANSCRIPT: ENST00000402961 |
| 17 | 75612921 | G | A | 222 | 42 | ENSG00000167291 | TBC1D16 | ENST00000310924 | INTRON |
| 16 | 67516490 | G | A | 52 | 19 | ENSG00000103047 | TMCO7 | ENST00000261778 | INTRON |
| 16 | 67516490 | G | A | 52 | 19 | ENSG00000203389 | AC009137.8 | ENST00000366212 | UPSTREAM: 3226 bases |
| 1 | 205164090 | G | A | 137 | 37 | ENSG00000162894 | FAIM3 | ENST00000367091 | UPSTREAM: 2124 bases |
| 1 | 205164090 | G | A | 137 | 37 | ENSG00000162896 | PIGR | ENST00000356495 | DOWNSTREAM: 4405 bases |
| 4 | 6726073 | G | C | 225 | 22 | ENSG00000170846 | AC093323.3 | ENST00000307533 | START_GAINED: CTG, UTR_5_PRIME: 768 bases from TSS |
| 2 | 132742596 | A | G | 89 | 62 |  |  |  | INTERGENIC |
| 1 | 205164060 | A | T | 177 | 32 | ENSG00000162894 | FAIM3 | ENST00000367091 | UPSTREAM: 2094 bases |
| 1 | 205164060 | A | T | 177 | 32 | ENSG00000162896 | PIGR | ENST00000356495 | DOWNSTREAM: 4435 bases |
| 11 | 68575088 | G | T | 77 | 16 | ENSG00000162341 | TPCN2 | ENST00000294309 | INTRON |
| 11 | 68575088 | G | T | 77 | 16 | ENSG00000162341 | TPCN2 | ENST00000356782 | INTRON |
| 16 | 48094914 | C | T | 82 | 25 | ENSG00000102935 | ZNF423 | ENST00000394716 | INTRON |
| 16 | 48094914 | C | T | 82 | 25 | ENSG00000102935 | ZNF423 | ENST00000262383 | INTRON |
| X | 2856702 | A | G | 124 | 19 | ENSG00000006756 | ARSD | ENST00000381154 | INTRON |
| X | 2856702 | A | G | 124 | 19 | ENSG00000006756 | ARSD | ENST00000217890 | INTRON |
| X | 2856702 | A | G | 124 | 19 | ENSG00000006756 | ARSD | ENST00000358177 | INTRON |
| 22 | 20745412 | T | C | 97 | 18 | ENSG00000217320 | IGLVIV-66-1 | ENST00000406817 | DOWNSTREAM: 2108 bases |
| 22 | 20745412 | T | C | 97 | 18 | ENSG00000220863 | IGLVV-66 | ENST00000402961 | UPSTREAM: 77 bases |
| 2 | 231610560 | T | C | 68 | 30 | ENSG00000204128 | C2orf72 | ENST00000373640 | UPSTREAM: 346 bases |
| 17 | 28173539 | A | G | 175 | 64 | ENSG00000176658 | MYO1D | ENST00000318217 | INTRON |
| 17 | 28173539 | A | G | 175 | 64 | ENSG00000176658 | MYO1D | ENST00000394649 | INTRON |
| 19 | 2526622 | T | C | 98 | 19 | ENSG00000176533 | AC005512.1-2 | ENST00000382159 | INTRON |
| 4 | 6726571 | A | G | 130 | 56 | ENSG00000170846 | AC093323.3 | ENST00000307533 | UTR_5_PRIME: 270 bases from TSS |
| 19 | 45664338 | C | T | 60 | 56 | ENSG00000090013 | BLVRB | ENST00000263368 | UPSTREAM: 821 bases |
| 19 | 45664338 | C | T | 60 | 56 | ENSG00000160460 | SPTBN4 | ENST00000344104 | UPSTREAM: 628 bases |
| 19 | 45664338 | C | T | 60 | 56 | ENSG00000160460 | SPTBN4 | ENST00000338932 | UPSTREAM: 628 bases |
| 19 | 45664338 | C | T | 60 | 56 | ENSG00000160460 | SPTBN4 | ENST00000352632 | UPSTREAM: 628 bases |
| 1 | 142123527 | T | C | 70 | 61 |  |  |  | INTERGENIC |
| 14 | 73074261 | A | G | 222 | 52 | ENSG00000187105 | HEATR4 | ENST00000334988 | INTRON |
| 14 | 73074261 | A | G | 222 | 52 | ENSG00000184227 | ACOT1 | ENST00000311148 | NON_SYNONYMOUS_CODING |
| 4 | 1787535 | A | C | 51 | 20 | ENSG00000168924 | LETM1 | ENST00000302787 | INTRON |
| 17 | 75612958 | G | C | 222 | 44 | ENSG00000167291 | TBC1D16 | ENST00000310924 | INTRON |
| 11 | 116189630 | A | G | 208 | 18 |  |  |  | INTERGENIC |
| 1 | 142123596 | A | C | 52 | 57 |  |  |  | INTERGENIC |
| 6 | 31347130 | A | G | 222 | 78 | ENSG00000204525 | HLA-C | ENST00000376237 | INTRON |
| 6 | 31347130 | A | G | 222 | 78 | ENSG00000204525 | HLA-C | ENST00000376235 | INTRON |
| 6 | 31347130 | A | G | 222 | 78 | ENSG00000204525 | HLA-C | ENST00000396254 | INTRON |
| 6 | 31347130 | A | G | 222 | 78 | ENSG00000204525 | HLA-C | ENST00000406626 | INTRON |
| 6 | 31347130 | A | G | 222 | 78 | ENSG00000214892 | AL671883.3-1 | ENST00000399193 | UPSTREAM: 4208 bases |
| 6 | 31347130 | A | C | 222 | 78 | ENSG00000204525 | HLA-C | ENST00000376237 | INTRON |
| 6 | 31347130 | A | C | 222 | 78 | ENSG00000204525 | HLA-C | ENST00000376235 | INTRON |
| 6 | 31347130 | A | C | 222 | 78 | ENSG00000204525 | HLA-C | ENST00000396254 | INTRON |
| 6 | 31347130 | A | C | 222 | 78 | ENSG00000204525 | HLA-C | ENST00000406626 | INTRON |
| 6 | 31347130 | A | C | 222 | 78 | ENSG00000214892 | AL671883.3-1 | ENST00000399193 | UPSTREAM: 4208 bases |
| 17 | 38756821 | T | C | 82 | 86 |  |  |  | INTERGENIC |
| 11 | 180025 | G | T | 68 | 38 | ENSG00000177951 | BET1L | ENST00000410108 | INTRON |
| 11 | 180025 | G | T | 68 | 38 | ENSG00000188076 | SCGB1C1 | ENST00000342878 | UPSTREAM: 3055 bases |
| 6 | 30002372 | C | G | 129 | 70 | ENSG00000219322 | HLA-K | ENST00000406831 | TRANSCRIPT: ENST00000406831 |
| 6 | 30002372 | C | G | 129 | 70 | ENSG00000181573 | HCG4P6 | ENST00000402127 | TRANSCRIPT: ENST00000402127 |
| 8 | 2063085 | A | T | 124 | 28 | ENSG00000036448 | MYOM2 | ENST00000262113 | INTRON |
| 17 | 75613002 | C | T | 163 | 40 | ENSG00000167291 | TBC1D16 | ENST00000310924 | INTRON |
| 17 | 75612949 | A | G | 191 | 49 | ENSG00000167291 | TBC1D16 | ENST00000310924 | INTRON |
| 17 | 76222913 | T | C | 78 | 10 | ENSG00000141564 | AC127496.5-2 | ENST00000306801 | INTRON |
| 17 | 38756838 | T | C | 96 | 90 |  |  |  | INTERGENIC |
| 2 | 231085968 | G | A | 119 | 20 | ENSG00000067066 | SP100 | ENST00000340126 | INTRON |
| 2 | 231085968 | G | A | 119 | 20 | ENSG00000067066 | SP100 | ENST00000264052 | INTRON |
| 2 | 231085968 | G | A | 119 | 20 | ENSG00000067066 | SP100 | ENST00000409112 | DOWNSTREAM: 4863 bases |

Table S5: Novel SNPs identified in lower control peaks than in ASH.

| **Chromosome** | **Position** | **Reference** | **Change** | **Quality** | **Coverage** | **Gene_ID** | **Gene_name** | **Trancript_ID** | **Effect** |
| --- | --- | --- | --- | --- | --- | --- | --- | --- | --- |
| 20 | 3167035 | G | A | 208 | 67 | ENSG00000088836 | SLC4A11 | ENST00000380056 | UPSTREAM: 662 bases |
| 20 | 3167035 | G | A | 208 | 67 | ENSG00000088836 | SLC4A11 | ENST00000380059 | UPSTREAM: 223 bases |
| 2 | 236741534 | G | A | 124 | 50 | ENSG00000168505 | GBX2 | ENST00000306318 | UPSTREAM: 143 bases |
| 7 | 158630474 | A | G | 120 | 18 | ENSG00000106018 | VIPR2 | ENST00000402066 | UPSTREAM: 162 bases |
| 7 | 158630474 | A | G | 120 | 18 | ENSG00000106018 | VIPR2 | ENST00000262178 | UPSTREAM: 64 bases |
| 11 | 63823686 | C | T | 168 | 65 | ENSG00000182450 | KCNK4 | ENST00000394525 | NON_SYNONYMOUS_CODING |
| 11 | 63823686 | C | T | 168 | 65 | ENSG00000219435 | C11orf20 | ENST00000328404 | UPSTREAM: 753 bases |
| 11 | 63823686 | C | T | 168 | 65 | ENSG00000207024 | Y_RNA | ENST00000384297 | DOWNSTREAM: 3500 bases |
| 11 | 63823686 | C | T | 168 | 65 | ENSG00000126432 | PRDX5 | ENST00000394513 | UPSTREAM: 4337 bases |
| 4 | 4439916 | G | A | 125 | 58 | ENSG00000168824 | AC110814.2 | ENST00000382740 | INTRON |
| 4 | 4439916 | G | A | 125 | 58 | ENSG00000168824 | AC110814.2 | ENST00000327590 | INTRON |
| 4 | 4439916 | G | A | 125 | 58 | ENSG00000168824 | AC110814.2 | ENST00000397958 | INTRON |
| 2 | 227738021 | G | C | 157 | 82 | ENSG00000169031 | COL4A3 | ENST00000304990 | INTRON |
| 2 | 227738021 | G | C | 157 | 82 | ENSG00000169031 | COL4A3 | ENST00000335583 | INTRON |
| 2 | 227738021 | G | C | 157 | 82 | ENSG00000169031 | COL4A3 | ENST00000396588 | INTRON |
| 2 | 227738021 | G | C | 157 | 82 | ENSG00000169031 | COL4A3 | ENST00000315699 | INTRON |
| 2 | 227738021 | G | C | 157 | 82 | ENSG00000169031 | COL4A3 | ENST00000396572 | INTRON |
| 2 | 227738021 | G | C | 157 | 82 | ENSG00000169031 | COL4A3 | ENST00000396574 | INTRON |
| 2 | 227738021 | G | C | 157 | 82 | ENSG00000169031 | COL4A3 | ENST00000396578 | INTRON |
| 2 | 227738021 | G | C | 157 | 82 | ENSG00000169031 | COL4A3 | ENST00000328380 | INTRON |
| 2 | 227738021 | G | C | 157 | 82 | ENSG00000081052 | COL4A4 | ENST00000329662 | UPSTREAM: 948 bases |
| 8 | 11909617 | C | T | 225 | 42 |  |  |  | INTERGENIC |
| 9 | 139331081 | G | A | 121 | 28 | ENSG00000187609 | EXD3 | ENST00000340951 | INTRON |
| 9 | 139331081 | G | A | 121 | 28 | ENSG00000187609 | EXD3 | ENST00000342129 | INTRON |
| 2 | 176696112 | C | T | 149 | 51 | ENSG00000218175 | RPLP1P4 | ENST00000405359 | INTRON |
| 2 | 176696112 | C | T | 149 | 51 | ENSG00000128710 | HOXD10 | ENST00000249501 | DOWNSTREAM: 3197 bases |
| 2 | 176696112 | C | T | 149 | 51 | ENSG00000128709 | HOXD9 | ENST00000249499 | NON_SYNONYMOUS_CODING |
| 2 | 176696112 | C | T | 149 | 51 | ENSG00000128709 | HOXD9 | ENST00000392535 | NON_SYNONYMOUS_CODING |
| 2 | 176696112 | C | T | 149 | 51 | ENSG00000175892 | AC009336.13 | ENST00000313266 | DOWNSTREAM: 4689 bases |
| 1 | 11461507 | C | T | 98 | 60 | ENSG00000204624 | PTCHD2 | ENST00000389575 | UPSTREAM: 375 bases |
| 1 | 11461507 | C | T | 98 | 60 | ENSG00000204624 | PTCHD2 | ENST00000294484 | UPSTREAM: 375 bases |
| 12 | 8434419 | C | A | 92 | 65 |  |  |  | INTERGENIC |
| 7 | 1674026 | G | A | 119 | 70 |  |  |  | INTERGENIC |
| 1 | 7663462 | G | T | 132 | 91 | ENSG00000171735 | CAMTA1 | ENST00000377565 | INTRON |
| 1 | 7663462 | G | T | 132 | 91 | ENSG00000171735 | CAMTA1 | ENST00000303646 | INTRON |
| 1 | 7663462 | G | T | 132 | 91 | ENSG00000171735 | CAMTA1 | ENST00000303635 | INTRON |
| 9 | 21549451 | C | T | 179 | 79 | ENSG00000171889 | AL353732.14 | ENST00000304425 | INTRON |
| 18 | 32131711 | G | T | 96 | 44 | ENSG00000134775 | FHOD3 | ENST00000359247 | START_GAINED: CTG, UTR_5_PRIME: 86 bases from TSS |
| 18 | 32131711 | G | T | 96 | 44 | ENSG00000134775 | FHOD3 | ENST00000257209 | START_GAINED: CTG, UTR_5_PRIME: 86 bases from TSS |
| 5 | 5193730 | C | T | 132 | 65 | ENSG00000145536 | ADAMTS16 | ENST00000274181 | INTRON |
| 1 | 224803886 | G | C | 225 | 44 | ENSG00000203685 | C1orf95 | ENST00000366789 | INTRON |
| 1 | 224803886 | G | C | 225 | 44 | ENSG00000203685 | C1orf95 | ENST00000366788 | INTRON |
| 17 | 58059377 | C | T | 225 | 61 | ENSG00000011028 | MRC2 | ENST00000303375 | INTRON |
| 2 | 192767935 | G | T | 119 | 41 | ENSG00000144339 | TMEFF2 | ENST00000392314 | UPSTREAM: 48 bases |
| 2 | 192767935 | G | T | 119 | 41 | ENSG00000144339 | TMEFF2 | ENST00000272771 | UPSTREAM: 31 bases |
| 2 | 192767935 | G | T | 119 | 41 | ENSG00000144339 | TMEFF2 | ENST00000409056 | UPSTREAM: 440 bases |
| 5 | 127901866 | A | G | 124 | 60 | ENSG00000138829 | FBN2 | ENST00000262464 | UPSTREAM: 232 bases |
| 19 | 2440445 | T | C | 163 | 52 |  |  |  | INTERGENIC |
| 2 | 132826566 | C | T | 117 | 63 |  |  |  | INTERGENIC |
| 19 | 10489087 | C | A | 225 | 76 | ENSG00000180739 | S1PR5 | ENST00000359134 | UTR_5_PRIME: 31 bases from TSS |
| 19 | 10489087 | C | A | 225 | 76 | ENSG00000180739 | S1PR5 | ENST00000333430 | UTR_5_PRIME: 31 bases from TSS |
| 9 | 33513922 | G | A | 84 | 86 | ENSG00000159712 | ANKRD18B | ENST00000290943 | UPSTREAM: 470 bases |
| 9 | 33513922 | G | A | 84 | 86 | ENSG00000159712 | ANKRD18B | ENST00000354752 | UPSTREAM: 512 bases |
| 1 | 238228099 | C | T | 155 | 56 |  |  |  | INTERGENIC |
| 7 | 158630540 | C | G | 164 | 31 | ENSG00000106018 | VIPR2 | ENST00000402066 | UPSTREAM: 228 bases |
| 7 | 158630540 | C | G | 164 | 31 | ENSG00000106018 | VIPR2 | ENST00000262178 | UPSTREAM: 130 bases |
| 1 | 238228059 | G | A | 119 | 60 |  |  |  | INTERGENIC |
| 6 | 37772824 | G | A | 186 | 61 | ENSG00000112139 | MDGA1 | ENST00000373401 | START_GAINED: ATG, UTR_5_PRIME: 259 bases from TSS |
| 6 | 37772824 | G | A | 186 | 61 | ENSG00000112139 | MDGA1 | ENST00000297153 | START_GAINED: ATG, UTR_5_PRIME: 259 bases from TSS |
| 5 | 127901820 | C | G | 126 | 74 | ENSG00000138829 | FBN2 | ENST00000262464 | UPSTREAM: 186 bases |
| 12 | 120501849 | G | A | 75 | 76 | ENSG00000089094 | FBXL10 | ENST00000377071 | INTRON |
| 12 | 120501849 | G | A | 75 | 76 | ENSG00000089094 | FBXL10 | ENST00000261824 | INTRON |
| 12 | 120501849 | G | A | 75 | 76 | ENSG00000089094 | FBXL10 | ENST00000397478 | INTRON |
| 12 | 120501849 | G | A | 75 | 76 | ENSG00000089094 | FBXL10 | ENST00000377069 | INTRON |
| 12 | 120501849 | G | A | 75 | 76 | ENSG00000089094 | FBXL10 | ENST00000397480 | INTRON |
| 8 | 104582658 | G | T | 106 | 33 | ENSG00000176406 | RIMS2 | ENST00000406091 | INTRON |
| 8 | 104582658 | G | T | 106 | 33 | ENSG00000176406 | RIMS2 | ENST00000329869 | INTRON |
| 8 | 104582658 | G | T | 106 | 33 | ENSG00000176406 | RIMS2 | ENST00000402998 | INTRON |
| 9 | 33667373 | G | C | 124 | 38 | ENSG00000147974 | AL356489.14-3 | ENST00000277031 | UPSTREAM: 724 bases |
| 15 | 71448249 | G | T | 174 | 62 | ENSG00000138622 | HCN4 | ENST00000261917 | UPSTREAM: 19 bases |
| 5 | 127901892 | G | C | 112 | 57 | ENSG00000138829 | FBN2 | ENST00000262464 | UPSTREAM: 258 bases |
| 7 | 107883042 | G | C | 85 | 59 | ENSG00000091129 | NRCAM | ENST00000379028 | INTRON |
| 7 | 107883042 | G | C | 85 | 59 | ENSG00000091129 | NRCAM | ENST00000379032 | INTRON |
| 7 | 107883042 | G | C | 85 | 59 | ENSG00000091129 | NRCAM | ENST00000379022 | INTRON |
| 7 | 107883042 | G | C | 85 | 59 | ENSG00000091129 | NRCAM | ENST00000379024 | INTRON |
| 7 | 107883042 | G | C | 85 | 59 | ENSG00000091129 | NRCAM | ENST00000351718 | INTRON |
| 10 | 42569543 | G | A | 128 | 75 |  |  |  | INTERGENIC |
| 8 | 118019832 | G | A | 135 | 61 | ENSG00000205002 | C8orf85 | ENST00000378279 | NON_SYNONYMOUS_CODING |
| 20 | 3167035 | G | A | 208 | 67 | ENSG00000088836 | SLC4A11 | ENST00000380056 | UPSTREAM: 662 bases |
| 20 | 3167035 | G | A | 208 | 67 | ENSG00000088836 | SLC4A11 | ENST00000380059 | UPSTREAM: 223 bases |
| 14 | 70345937 | G | T | 225 | 48 | ENSG00000006432 | MAP3K9 | ENST00000005198 | UPSTREAM: 296 bases |
| 14 | 70345937 | G | T | 225 | 48 | ENSG00000006432 | MAP3K9 | ENST00000381250 | UPSTREAM: 296 bases |
| 2 | 112529253 | C | T | 57 | 27 | ENSG00000153214 | TMEM87B | ENST00000283206 | UPSTREAM: 32 bases |
| 9 | 15296220 | C | G | 177 | 47 | ENSG00000155158 | TTC39B | ENST00000380849 | INTRON |
| 9 | 15296220 | C | G | 177 | 47 | ENSG00000155158 | TTC39B | ENST00000355694 | INTRON |
| 9 | 15296220 | C | G | 177 | 47 | ENSG00000155158 | TTC39B | ENST00000380850 | INTRON |
| 1 | 67292023 | C | A | 225 | 100 | ENSG00000116704 | SLC35D1 | ENST00000235345 | INTRON |
| 8 | 144695161 | G | T | 98 | 99 | ENSG00000208449 | AC067930.7-1 | ENST00000385714 | UPSTREAM: 262 bases |
| 8 | 144695161 | G | T | 98 | 99 | ENSG00000221399 | 7SK | ENST00000408472 | UPSTREAM: 262 bases |
| 8 | 144695161 | G | T | 98 | 99 | ENSG00000014164 | ZC3H3 | ENST00000262577 | UPSTREAM: 398 bases |
| 10 | 69837050 | C | T | 100 | 96 | ENSG00000204130 | RUFY2 | ENST00000399200 | NON_SYNONYMOUS_CODING |
| 10 | 69837050 | C | T | 100 | 96 | ENSG00000204130 | RUFY2 | ENST00000388768 | UPSTREAM: 98 bases |
| 10 | 69837050 | C | T | 100 | 96 | ENSG00000204130 | RUFY2 | ENST00000342616 | UPSTREAM: 10 bases |
| 6 | 42016447 | G | A | 99 | 84 | ENSG00000112576 | CCND3 | ENST00000372991 | INTRON |
| 6 | 42016447 | G | A | 99 | 84 | ENSG00000112576 | CCND3 | ENST00000372987 | INTRON |
| 6 | 42016447 | G | A | 99 | 84 | ENSG00000112576 | CCND3 | ENST00000372988 | INTRON |
| 10 | 70151660 | T | G | 225 | 91 | ENSG00000060339 | CCAR1 | ENST00000265872 | INTRON |
| 20 | 3818023 | G | A | 127 | 93 | ENSG00000125779 | PANK2 | ENST00000316562 | SYNONYMOUS_CODING |
| 20 | 3818023 | G | A | 127 | 93 | ENSG00000125779 | PANK2 | ENST00000336066 | INTRON |
| 20 | 3818023 | G | A | 127 | 93 | ENSG00000125779 | PANK2 | ENST00000399552 | UPSTREAM: 440 bases |
| 20 | 3818023 | G | A | 127 | 93 | ENSG00000125779 | PANK2 | ENST00000361370 | UPSTREAM: 39 bases |
| 17 | 27795629 | G | T | 161 | 85 | ENSG00000108671 | PSMD11 | ENST00000261712 | UTR_5_PRIME: 26 bases from TSS |
| 7 | 100110959 | C | T | 225 | 98 | ENSG00000172354 | GNB2 | ENST00000393926 | UTR_5_PRIME: 130 bases from TSS |
| 7 | 100110959 | C | T | 225 | 98 | ENSG00000172354 | GNB2 | ENST00000303210 | INTRON |
| 7 | 100110959 | C | T | 225 | 98 | ENSG00000146830 | GIGYF1 | ENST00000275732 | DOWNSTREAM: 4107 bases |
| 7 | 100110959 | C | T | 225 | 98 | ENSG00000172354 | GNB2 | ENST00000393924 | UPSTREAM: 738 bases |
| 2 | 112529253 | C | T | 57 | 27 | ENSG00000153214 | TMEM87B | ENST00000283206 | UPSTREAM: 32 bases |
| 2 | 113119870 | C | G | 87 | 45 | ENSG00000144136 | SLC20A1 | ENST00000272542 | UPSTREAM: 128 bases |
| 4 | 83514200 | G | A | 178 | 95 | ENSG00000138668 | HNRNPD | ENST00000307213 | UPSTREAM: 345 bases |
| 4 | 83514200 | G | A | 178 | 95 | ENSG00000138668 | HNRNPD | ENST00000313899 | UPSTREAM: 27 bases |
| 4 | 83514200 | G | A | 178 | 95 | ENSG00000138668 | HNRNPD | ENST00000353341 | UPSTREAM: 27 bases |
| 4 | 83514200 | G | A | 178 | 95 | ENSG00000138668 | HNRNPD | ENST00000352301 | UPSTREAM: 27 bases |
| 8 | 142080475 | C | G | 85 | 69 | ENSG00000169398 | PTK2 | ENST00000354438 | UTR_5_PRIME: 731 bases from TSS |
| 8 | 142080475 | C | G | 85 | 69 | ENSG00000169398 | PTK2 | ENST00000340930 | UTR_5_PRIME: 189 bases from TSS |
| 8 | 142080475 | C | G | 85 | 69 | ENSG00000169398 | PTK2 | ENST00000395218 | UTR_5_PRIME: 189 bases from TSS |
| 20 | 34101144 | G | A | 167 | 42 |  |  |  | INTERGENIC |
| 18 | 3441606 | C | A | 151 | 97 | ENSG00000177426 | TGIF | ENST00000343820 | INTRON |
| 18 | 3441606 | C | A | 151 | 97 | ENSG00000177426 | TGIF | ENST00000401449 | INTRON |
| 18 | 3441606 | C | A | 151 | 97 | ENSG00000177426 | TGIF | ENST00000345133 | INTRON |
| 18 | 3441606 | C | A | 151 | 97 | ENSG00000177426 | TGIF | ENST00000405385 | INTRON |
| 18 | 3441606 | C | A | 151 | 97 | ENSG00000177426 | TGIF | ENST00000407501 | INTRON |
| 18 | 3441606 | C | A | 151 | 97 | ENSG00000177426 | TGIF | ENST00000340165 | INTRON |
| 18 | 3441606 | C | A | 151 | 97 | ENSG00000177426 | TGIF | ENST00000330513 | UTR_5_PRIME: 372 bases from TSS |
| 18 | 3441606 | C | A | 151 | 97 | ENSG00000177426 | TGIF | ENST00000400167 | UPSTREAM: 2166 bases |

Table S6: Primers

| USF1 primers |  |  |
| --- | --- | --- |
| Primer Name | Forward Primer | Reverse Primer |
| USF1_1 | CTAGCAACCAGGAGCCAAAT | CGGCGTAATCTCCCACTG |
| USF1_2 | CTCAACAGACCCAGCGATTT | GCTTAGGGCTGACTGCAAAC |
| USF1_3 | TGGCTGAAACTCACATCCAG | TCAACAATCCACCCCTGAGT |
| USF1_4 | GACTTCCGCCCCTGACTC | GGGCTCTTGCGTCATCAG |
| USF1_5 | AGGACTTCCCTCCGACAAAG | CCCTAGGTGGAAACCCCATA |
| USF1_6 | AAGGGAAAGGCTAGCTCCAG | AAGCTCCGAGCCTCTTCATT |
| USF1_7 | CAGCAAGGACGCCATCTT | GGTGGGAAACCTAACGTCAA |
| USF1_8 | GCGACAGGGTAGCCATTAAA | TCTGTGATTCGGCAGAAATG |
| USF1_9 | GCAGGCTGTTAAATCCCACT | ACGTGGCCACTGTCTACCTC |
| USF1_10 | GCCCAGTGTTTGACTATTACCC | CCATGGCACTGAAAATGG |
| USF1_11 | GCGTCTATACAGCGTGTTGC | ATGGGACCCTGCCCTTTT |
| USF1_12 | TGTGTATCGCTGCACCACAT | ACTGCGAGTGTTTCCTCTGG |
| USF1_13 | CAGCAAGGACGCCATCTT | CGTAGGAAGTCGTAGGGAAGG |
|  |  |  |
| **mRNA primers for USF1** |  |  |
| ADK_mRNA | ttgtcaattatgccaaataatctct | tgtgtccaattacagagttcca |
| COPZ1_mRNA | acaagacccatcggactgac | cataggagctgccaatcaca |
| OSBPL6_mRNA | cactgtctcaggctggtgag | tgacgaacttgcagagagga |
| ANAPC5_mRNA | cttttgcgcagctcattctt | ccactaagaacatggcacga |
| NEU1_mRNA | ctgatgaatgccagccctat | agggtcgaaggtcacatcac |
| SNX16_mRNA | accgggtccatttgatagc | ttccatctccttttgtttttcaa |
| SUPV3L1_mRNA | atatggcagtctcccacctg | ccatgccaattgcatctgta |
| PPARg_mRNA | aaggccattttctcaaacga | tcaaaggagtgggagtggtc |
| ARSK_mRNA | cccttcaccatcttctggag | acagggtgcatttctgacaa |
| DYRK1B_mRNA | ctgcgtaagctctctgtgga | ccttcttgttgctcgaatcc |
|  |  |  |
| **mRNA primers for Histone modifications** |  |  |
| TCEB3_mRNA_ | aacgaaatgctgagcctgat | tggtcagggctataggatcg |
| IL6R_mRNA | ctcctgccagttagcagtcc | atcaggctgcaagattccac |
| HNF4a_mRNA | caggctcaagaaatgcttcc | ggctgctgtcctcatagctt |
| IL15RA_mRNA | agccaagaactgggaactca | aggacagtggacgtggagat |
| ALAS1_mRNA | aaaactgccccaagatgatg | ctgttggaccttggccttag |
| IFITM3_mRNA | atgtcgtctggtccctgttc | gccaaccatcttcctgtcc |
| GYG1_mRNA | ctgacaaagctccactgctg | tctggtgctgctgacaattc |
| ATAD2B_mRNA | tcagcgagaggaatggaact | atggcttcgaagggataagc |
| IGFBP1_mRNA | tatgatggctcgaaggctct | cctgtgccttggctaaactc |
| FAIM3_mRNA | ggggaaagacccagaaagtc | tatgcaggcatctggaacaa |
| PRKAR1B_mRNA | acctggatgacaacgagagg | acacatccacttccccttga |
| DDX3X_mRNA | caaaaccactcccaccaagt | cctgttgcctcaactggaat |
| PDK1_mRNA | tcaccaggacagccaataca | acctctgttggcatggtgtt |
| ARL6IP4_mRNA | CCTGACGGATGAGCAGAAGT | ACCTCGCCATCTCCCTTAAT |
| AMD1_mRNA | GGTACCACCCTCTTGCTGAA | TGGGTACCCTTGGTGAGAAG |
| DYRK1B_mRNA | ctgcgtaagctctctgtgga | ccttcttgttgctcgaatcc |
| ELP3_mRNA | ctggcacttgcaagaatgaa | tggtatggccgtactttgtg |
| GAPDH_mRNA | GAAGGTGAAGGTCGGAGTCAAC | CAGAGTTAAAAGCAGCCCTGGT |
